# Multifunctional Au–Ag–Cr Nanocomposites: From Multiplex Sensing and Advanced Logic Computing to Scalable Information Protection

**DOI:** 10.34133/research.0763

**Published:** 2025-07-22

**Authors:** Jie Zhou, Jiao Yang Lu, Zhi Xin Xie, Dong Hua Wang, Bin Sheng He, Wei Tao Huang

**Affiliations:** ^1^ Hunan Key Laboratory of the Research and Development of Novel Pharmaceutical Preparations, Hunan Provincial University Key Laboratory of the Fundamental and Clinical Research on Functional Nucleic Acid, “The 14th Five-Year Plan” Application Characteristic Discipline of Hunan Province (Clinical Medicine), School of Nursing, Changsha Medical University, Changsha 410219, P. R. China.; ^2^ State Key Laboratory of Developmental Biology of Freshwater Fish, Hunan Provincial Key Laboratory of Microbial Molecular Biology, College of Life Science, Hunan Normal University, Changsha 410081, P. R. China.

## Abstract

Polymetallic nanomaterials have important development potential by integrating the properties and advantages of their components. However, their preparation paradigm urgently needs to be updated to make full use of their characteristics to realize their multifaceted applications. In this study, multifunctional trimetallic gold–silver–chromium nanocomposites (Au–Ag–Cr NCs) were successfully synthesized, and their polymetallic plasmonic absorption properties were used in a variety of applications, from multimode and multianalyte sensing, advanced arithmetic, and reversible logic to long-text information protection. Au–Ag–Cr NCs with Au or Ag nanoparticles anchored on Cr nanobelts were synthesized using Au–Cr nanoseeds, Ag^+^, ascorbic acid as the reductant, and sodium dodecylbenzenesulfonate as the stabilizer. The resulting Au–Ag–Cr NCs exhibited multichannel sensing ability for 2 analytes (Hg^2+^ and hypochlorite), with remarkably improved selectivity and sensitivity when analyzing combined channels. Multiresponsiveness of Au–Ag–Cr NCs to diverse substance combinations underpins input–output mapping relationships, enabling advanced molecular logic computations (such as arithmetic and reversible logic). By digitizing its intrinsic sensing and response mechanisms, advanced molecular information protection (including encoding, encryption, and information concealment) for extended text were successfully implemented. This work not only offers novel insights into the design and multifunctionality of multicomponent nanocomposites but also paves the way for a new paradigm in molecular information technology that integrates sensing, logic processing, and information security.

## Introduction

Multimetallic nanomaterials (MNMs) are a type of composite material composed of 2 or more metal components, which have garnered widespread attention due to their unique physical, chemical, optical, and electrical properties [1,2]. These materials combine the characteristics and advantages of each component, exhibiting marked synergistic effects that make them superior in performance to monometallic nanomaterials and introducing new performance features [1,3,4]. For example, bimetallic nanomaterials incorporating noble metals (e.g., Au–Ag, Pt–Pd, and Au–Pt) have become research hotspots due to their excellent catalytic performance and optical properties [5–7]. With the deepening of research, trimetallic nanomaterials, as an extension of bimetallic nanomaterials, further enhance the performance of the materials and broaden their application scope by introducing a third metal element [8,9]. Because of their compositional and structural diversity, these materials exhibit more complex physicochemical properties, providing new possibilities for applications in fields such as sensing [10], catalysis [11], and information science. The expansion of these applications not only relies on the properties of the materials themselves but also closely relates to their preparation methods. Currently, the main preparation methods for MNMs include seed-mediated growth method [11], multimetal diffusion strategy [12], and guest species encapsulation strategy [1,13]. Among these, seed-mediated growth method, as a bottom-up construction strategy, is widely used because of its multifunctionality, simplicity, and convenience. However, given the complexity of different metal component combinations and their effects on material structure and performance, proposing new manufacturing paradigms is particularly important. This will not only accelerate the development process of novel MNMs but also support innovation in related application scenarios.

Life, as a complex individual, exhibits highly intricate molecular interactions and biochemical reaction networks [14]. Biological systems in nature, especially bees, ants, and birds, provide valuable insights through molecular encoding and transmission of information, such as pheromones [15]. Inspired by these powerful collaborations and the intricate operations within complex molecular networks, scientists are actively pursuing the development new artificial molecules and nanosystems to achieve efficient sensing, programmable control, and advancements in molecular information technology (MIT) [16]. As an emerging frontier, MIT spans diverse areas, including molecular information processing (such as logic computation [17]), information storage (e.g., DNA data storage [18]), and information security [19]. The rapid development of these technologies relies on the scalable design and stimulus-responsive characteristics of molecular or nanosystems. Existing research has successfully demonstrated the potential of these systems in various applications, including sensing, logic computation [20,21], and information protection. By combining and sequencing input and output operations, molecular logic devices can be constructed, such as basic logic gates [22–24], fuzzy logic [25–27], half adders, full adders, parity generators/checkers, keypad locks [28], and even reversible logic [29], to meet the growing demand for computational complexity. Harnessing simple molecular or nanosystems to construct large-scale parallel logic circuits and execute complex logic operations has emerged as a promising research direction. For example, by tapping into the multichannel and multisubstance sensing capabilities of these systems can substantially augment the number of logic inputs and outputs, thereby expanding the scope of logic operations [22–24]. Meanwhile, an emerging area of interest is the development or utilization of molecular or nanosystems to explore their intrinsic molecular information characteristics and achieve information protection, such as encryption and concealment. Recently, some organic molecules [30–32], polymers [33], biomolecules [34], and nanomaterials [22,35] have been creatively used for encoding, encrypting, and hiding information for data storage [33,36,37] and security (e.g., molecular keypad locks [38,39], authentication, cryptography, and steganography) [32,40–44]. The intrinsic chemical properties (such as color, absorption, or fluorescence spectra) [32,41] and unique structures (e.g., polymer sequences or DNA) [33,34] of these (bio)molecules offer potential for developing multilayer security technologies that encompass access control, information encryption, and information concealment [40]. Nonetheless, there is still ample room for research in the in-depth study of the properties and impacts of different components in nanomaterials, the exploration of diverse updates to nanomaterial preparation paradigms, and the application of molecular informatization beyond sensing functions. Innovations in expanding new stimulus–response mapping relations and developing novel molecular information coding paradigms, including the utilization of molecular features, relationships, and response signals, remain limited. These areas not only point to new directions for future technological development but also provide fertile ground for exploring more possibilities.

**Fig. 1.**
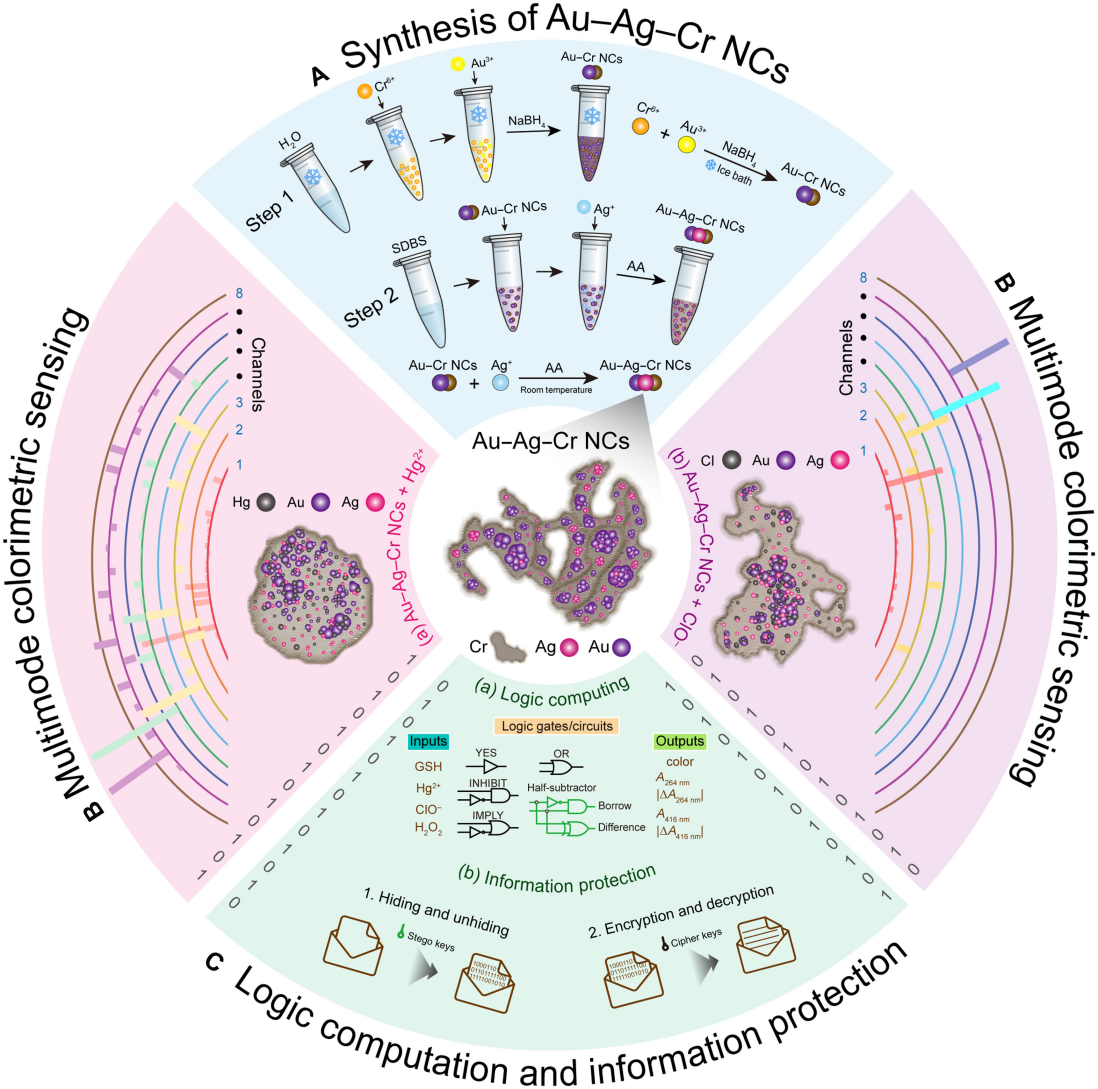
Schematic illustration of synthesis of multifunctional trimetallic Au–Ag–Cr NCs (A) for multimode and multianalyte sensing (B) and advanced arithmetic and reversible logic [C(a)], long-text information protection [C(b)].

Here, we report the synthesis of multifunctional trimetallic gold–silver–chromium nanocomposites (Au–Ag–Cr NCs) using a simple, rapid, and efficient approach. These NCs are utilized for multimode and multianalyte sensing, advanced arithmetic and reversible logic operations, and long-text information protection (Fig. 1). Using the Au–Cr nanoseed method, ascorbic acid (AA) reductant, and sodium dodecylbenzenesulfonate (SDBS) stabilizer at room temperature, trimetallic Au–Ag–Cr NCs were prepared and characterized by the distribution of Au/Ag nanoparticles (NPs) on the chromium nanobelts (Cr NBs; Fig. 1A). On the basis of the color, Au/Ag plasmonic absorption, and its combined signal changes, Au–Ag–Cr NCs enable highly sensitive and selective multimode colorimetric sensing of Hg^2+^ and hypochlorite (ClO^−^), even in real water samples. This can be attributed to the fact that Hg^2+^ and ClO^−^ etch the Au/Ag NPs in Au–Ag–Cr NCs (Fig. 1B). Compared to individual signal channels [such as absorption difference at 530 nm (Δ*A*_530 nm_)], by skillfully integrating multiple signal channels (such as Δ*A*_416 nm_ × Δ*A*_530 nm_), we have significantly improved the selectivity [at most 3.32-fold (12.03 time of Δ*A*_416 nm_ × Δ*A*_530 nm_:3.62 times of Δ*A*_530 nm_)] and sensitivity [at most 2,166.7-fold (39 nM Δ*A*_416 nm_:0.018 nM Δ*A*_416 nm_ × Δ*A*_700 nm_)] for Hg^2+^ [Fig. 1B(a)], as well as the selectivity [at most 5.86-fold (43.37 times of Δ*A*_416 nm_ × Δ*A*_700 nm_:7.40 times of Δ*A*_416 nm_)] and sensitivity [at most 1,653.8-fold (43 nM Δ*A*_416 nm_:0.026 nM Δ*A*_416 nm_ × Δ*A*_700 nm_)] for ClO^−^ [Fig. 1B(b)]. Furthermore, Au–Ag–Cr NCs respond to the addition of different substances as input signals and produce multimodal colorimetric responses as outputs, providing new possibilities for the development of batch and parallel multifunctional molecular logic, including basic gates, arithmetic, and reversible logic [Fig. 1C(a)]. Building on this, by binary converting and encoding the inherent responses of Au–Ag–Cr NCs, we have achieved molecular-level encryption and steganography techniques for long text based on molecular logic operations and multicoding selective responses, greatly enhancing the security and confidentiality of information [Fig. 1C(b)]. This research not only paves new avenues for the customized synthesis and diverse applications of multiple-metal NCs but also offers a fresh perspective and opportunities for deep investigation of molecular or nanoscale information processing and protection technologies to achieve more powerful functionality.

## Results and Discussion

### Preparation and characterization of Au–Ag–Cr NCs

**Fig. 2.**
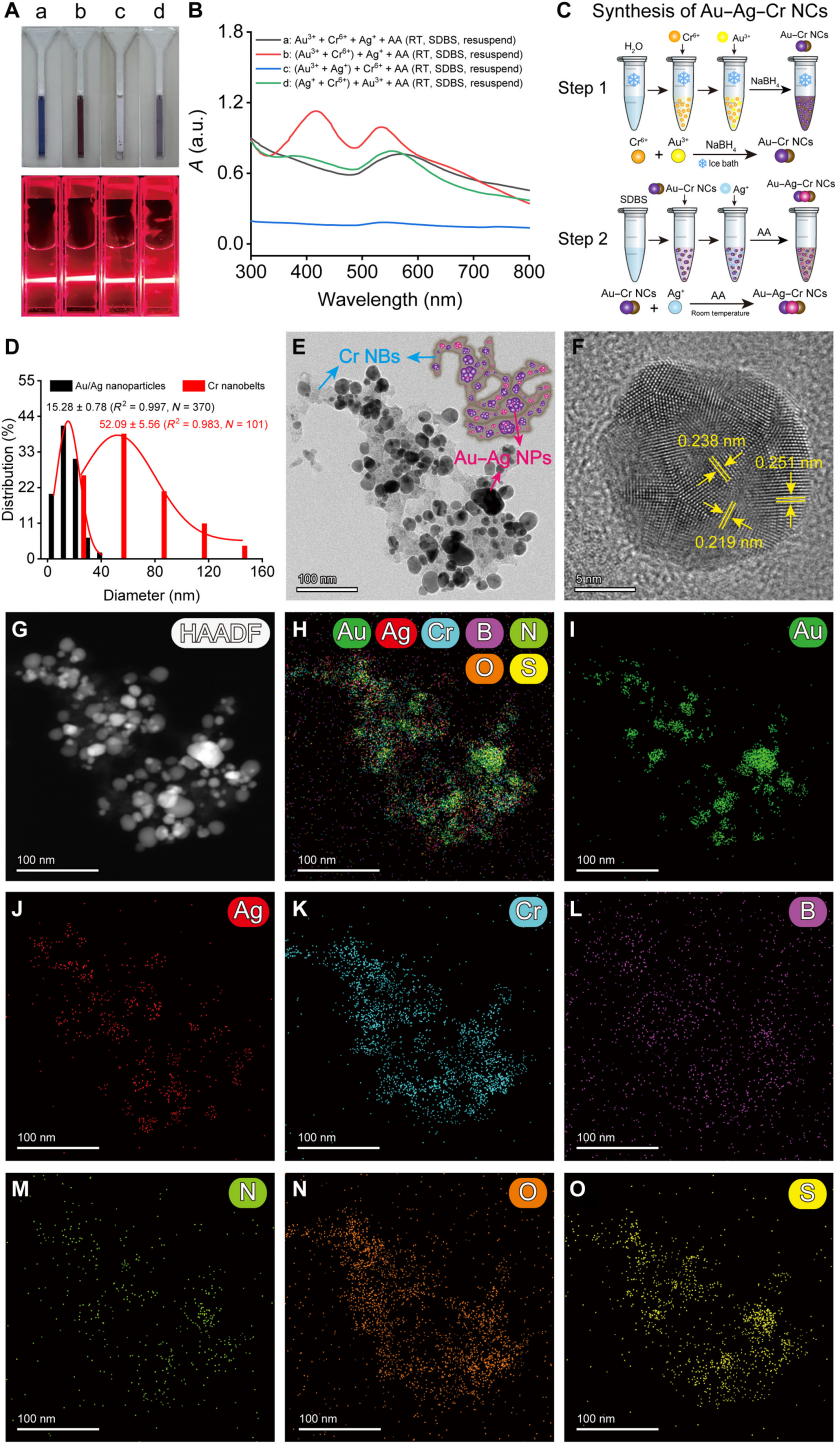
Preparation, TEM imaging, and EDS element mapping characterization of Au–Ag–Cr NCs. (A and B) The color and Tyndall effect photos (A) and absorption spectra (B) of resuspension of the reaction solution with different combinations using one-pot reduction and nanoseed methods at room temperature (RT). Au^3+^, 0.25 mM; Cr^6+^, 0.5 mM; Ag^+^ and AA, 1.75 mM; SDBS, 4 mM. Note that this concentration ratio is not the final optimized result. (C) Schematic diagram of the synthesis of Au–Ag–Cr NCs. (D) Statistical histograms of the diameters of Cr NBs (*N* = 101) and spherical Au/Ag NPs (*N* = 370) obtained by processing TEM images using ImageJ software. (E and F) TEM image (E) of the light brown colloidal solution [corresponding to group b in (A) and (B)] and its high-resolution TEM analysis for lattice fringes (F). (G to O) High-angle annular dark-field scanning TEM (HAADF-STEM) image (G) and EDS element mapping (H to O) of the light-brown colloidal solution. Scale bars, 100 and 5 nm.

To prepare Au–Ag–Cr NCs, we explored the effects of 2 methods—one-pot reduction and nanoseed-mediated growth—on the mixed reaction of 3 metal ions (Au^3+^, Ag^+^, and Cr^6+^) at different reaction temperatures (room temperature and 50 °C heating), different reducing agents (AA or NaBH_4_), and different stabilizers (SDBS). To evaluate the synthesis efficacy of Au–Ag–Cr NCs, we documented and analyzed the color, Tyndall effect, and absorption spectra of the mixed reaction solutions from various combinations (Figs. S1 to S4). Note that the initial concentrations of the various additives were unified as follows: Au^3+^, 0.25 mM; Cr^6+^, 0.5 mM; Ag^+^ and AA, 1.75 mM; and SDBS, 4 mM. These concentrations were then individually optimized in subsequent experiments. Under many combined conditions, only the Au–Cr nanoseed method (that is, Au–Cr NCs were first prepared by the reaction of Au^3+^ and Cr^6+^ ions with NaBH_4_), with the AA reducing agent and the SDBS stabilizer at room temperature can produce a stable, uniform colloidal solution with characteristic absorption peaks of both gold (around 536 nm) and silver (around 416 nm) nanomaterials [Fig. 2A(b) and B(b)]. For the direct mixing of Au^3+^, Ag^+^, Cr^6+^, Au–Ag nanoseeds, or Ag–Cr nanoseeds, the mixing solution prepared at room temperature with the AA reductant and SDBS stabilizer is either a clear solution with no characteristic absorption peak or only a single absorption peak of gold nanomaterial [Fig. 2A(a, c, and d) and B(a, c, and d)].

For at room temperature or 50 °C heating, AA reductant, and no stabilizer (Fig. S1), the characteristic absorption peaks of gold (about 530 nm) and silver (about 416 nm) nanomaterials can be also obtained only by the Au–Cr nanoseed method [Fig. S1I(b) and R(b)]. However, the resulting solution is not stably dispersed and precipitates after standing for a period of time (Fig. S1B and K). When a stabilizer SDBS was added to the reaction on the basis of the previous one (Fig. S2), the stability of the colloid solution was significantly improved [Fig. S2B, I(b), K, and R(b)]. However, when AA was replaced with NaBH_4_ (Figs. S3 and S4), characteristic absorption peaks of gold and silver NPs obviously appeared in the colloidal solution by the Au–Cr nanoseed method only at 50 °C [Figs. S3 and S4R(b)]. Similarly, without stabilizer SDBS, the colloidal solution is prone to rapid precipitation (Fig. S3B and K); while adding stabilizer SDBS, the stability of solution is improved (Fig. S4B and K). However, compared with NaBH_4_, AA can promote the reaction to obtain more NCs because its absorption peaks of Au NPs and Ag NPs were higher [Figs. S2I(b) and S4I(b)]. Subsequently, we monitored the time-dependent process of synthesizing Au–Ag–Cr NCs using the Au–Cr nanoseed method (Figs. S5 to S7). When AA was used as the reducing agent without SDBS, the mixed solution reacted completely in about 30 min with sedimentation starting after 70 min, accompanied by a decrease in characteristic absorption peak values (Fig. S5). In the presence of SDBS, the solution stabilized between 30 and 90 min, showing no significant color changes after 70 min (Fig. S6). In contrast, when NaBH_4_ was used as the reducing agent along with SDBS, the mixed solution required 270 min to maintain stable color, while the characteristic absorption peak value of the Ag NPs reached a maximum and then decreased (Fig. S7). The above results proved that the reducing agent AA could promote the synthesis of Au–Ag–Cr NCs at room temperature, and the presence of the stabilizer SDBS made the colloidal solution more stable. Therefore, we selected to prepare Au–Ag–Cr NCs using the Au–Cr nanoseed method (step 1) under at room temperature and AA reductant and SDBS stabilizer conditions (step 2; Fig. 2C).

Furthermore, we explored the optimal addition concentration by successively changing the concentrations of Ag^+^, AA, and SDBS and gradually increasing the concentration along a gradient of 0.25 mM. As shown in Fig. S8, when the concentration of AA (2 mM) and SDBS (3 mM) does not change and the concentration of Ag^+^ is 1.75 mM, the solution is the deepest color and has the highest absorption value at 416 and 530 nm. Similarly, following the above steps, the concentrations of AA and SDBS were changed along the gradient. When the AA concentration is 1.25 mM (Fig. S9), the color of the solution is darker, and the 2 characteristic absorption peaks in the absorption spectrum are high (considering that the AA concentration is 2 mM, the characteristic peak uplift of Ag NPs and Au NPs is not very large, and the position of the peak gradually shifts greatly; in the idea of saving cost, the added concentration of AA is finally selected as 1.25 mM). As shown in Fig. S10, when the SDBS concentration was 3.75 mM, the solution was deepest and the 2 characteristic absorption peaks reached their highest. The characteristic absorption peaks of the subsequent bulk prepared materials are stabilized at around 416 and 530 nm. Storage stability experiments found that although the absorption of Au–Ag–Cr NC colloidal solution showed a relatively significant change after 10 h of the reaction, it remained basically stable for 42 d (which is the point we have temporarily detected) thereafter (Fig. S11). Therefore, we conducted all relevant follow-up experiments using Au–Ag–Cr NCs after 10 h of preparation reaction to ensure their reliability and reproducibility in practical applications.

The transmission electron microscope (TEM) characterization results showed that the obtained light-brown colloidal solution mainly consisted of band-like Cr NBs [with an average diameter of 56.93 ± 2.77 (*R*^2^ = 0.990, *N* = 101); Fig. 2D] on which spherical Au/Ag NPs [with an average diameter of 14.15 ± 0.63 (*R*^2^ = 0.994, *N* = 370); Fig. 2D] were distributed (Fig. 2E). High-resolution TEM analysis revealed clear lattice fringes in the Au–Ag–Cr NCs, with average spacings of 0.219, 0.238, and 0.251 nm corresponding to the Ag(111) or Au(111) planes and the Ag(200) plane [45–47], respectively (Fig. 2F). Energy-dispersive spectrometer (EDS) element mapping confirmed the presence of Au, Ag, Cr, B, N, O, and S in the synthesized Au–Ag–Cr NCs (Fig. 2G to O). The green Au NPs and red Ag NPs are dotted on the branched band-like Cr NBs (Fig. 2H to J). Because Au–Ag–Cr NCs were prepared by the Au–Cr nanoseed method, the Ag NPs were uniformly dispersed on the Au–Cr nanostructures. The B and N elements were relatively distributed throughout the field of view (Fig. 2L and M), attributed to a small amount of residual by-products in the Au–Ag–Cr NCs. The uniform distribution of O and S elements across the entire surface of the Au–Ag–Cr NCs indicates that SDBS is stably adsorbed on the NCs (Fig. 2N and O).

**Fig. 3.**
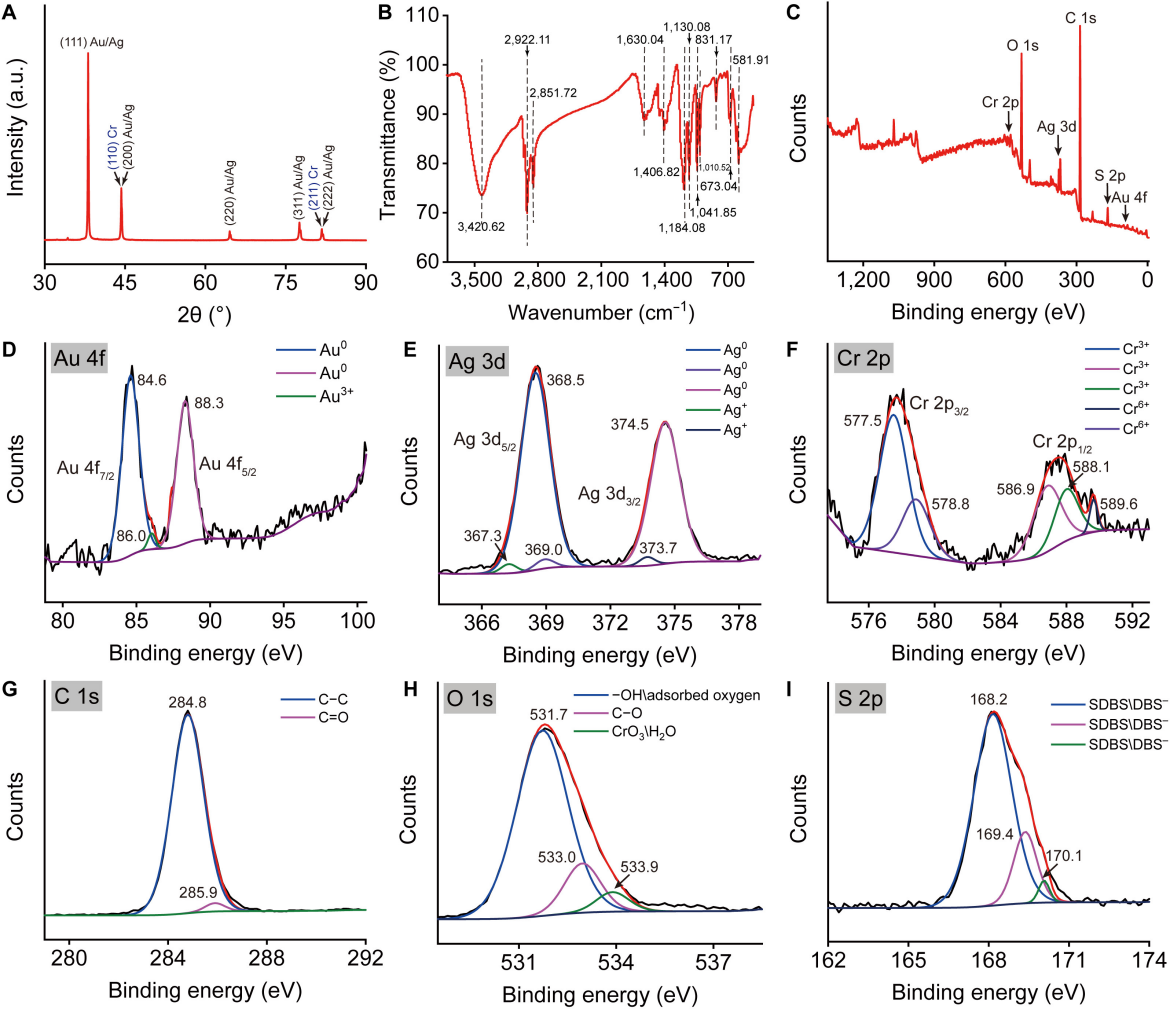
Characterization of Au–Ag–Cr NCs by XRD, FTIR, and XPS. (A and B) XRD pattern (A) and FTIR spectrum (B) of Au–Ag–Cr NCs. (C to F) XPS survey spectra (C) and core-level spectra of Au 4f (D), Ag 3d (E), Cr 2p (F), C 1s (G), O 1s (H), and S 2p (I) of Au–Ag–Cr NCs.

X-ray diffractometer (XRD) analysis revealed diffraction peaks at 2θ = 38.10°, 44.30°, 64.54°, 77.56°, and 81.76° corresponding to the (111), (200), (220), (311), and (222) planes of Au or Ag [47–49], respectively, while the diffraction peaks at 2θ = 44.30° and 81.76° also corresponded to the (110) and (211) planes of Cr (Fig. 3A) [50,51]. Fourier transform infrared spectrometer (FTIR) analysis in Fig. 3B indicated that the vibrational peak at 3,420.62 cm^−1^ was attributed to the O–H stretching of water molecules present in the NCs [52]. The peaks at 2,922.11 and 2,851.72 cm^−1^ were attributed to the asymmetric and symmetric stretching of –CH(CH_2_) groups [53,54], respectively. The peaks at 1,630.04 and 1,406.82 cm^−1^ corresponded to the C═O stretching vibration of carboxyl groups and the OH bending of carboxylate [55,56], respectively. In addition, the peaks at 1,184.08, 1,130.08, and 1,041.85 cm^−1^ corresponded to the S═O stretching of sulfonate –SO_3_– groups [57]. Because of interatomic vibrations, the FTIR peaks of oxides appeared in the region below 1,000 cm^−1^; [58] Hence, the peak at 831.17 cm^−1^ was associated with the Ag–O–C bonding [59]. The spectrum did not show a peak around 753 cm^−1^ for Ag–O, indicating that Ag may be not oxidized in NCs [60]. The peaks at 673.04 and 581.91 cm^−1^ corresponded to the stretching vibrations of Cr–O in Cr_2_O_3_ [61–63].

X-ray photoelectron spectrometer (XPS) survey spectrum showed that the Au–Ag–Cr NCs revealed 6 primary peaks corresponding to Cr 2p, O 1s, Ag 3d, C 1s, S 2p, and Au 4f (Fig. 3C). The high-resolution Au 4f core-level spectrum exhibited 2 main peaks at 84.6 eV (Au 4f_7/2_) and 88.3 eV (Au 4f_5/2_), with a splitting energy of 3.7 eV between them, indicative of metallic Au in its zero-valent state [64,65]. A secondary peak at 86.0 eV indicated the presence of trace amounts of gold in the form of Au(III) (Fig. 3D) [66,67]. The high-resolution core-level spectrum of Ag 3d exhibited 4 peaks, which could be deconvoluted into Ag 3d_5/2_ (368.5 eV) and Ag 3d_3/2_ (374.5 eV) [68,69], with a splitting energy of 6.0 eV, indicating the presence of metallic Ag [70]. In addition, 3 secondary peaks located at 367.3, 369.0, and 373.7 eV could be attributed to Ag_2_O (Fig. 3E) [71]. The high-resolution core-level spectrum of Cr 2p included 5 deconvoluted peaks at 577.5, 578.8, 586.9, 588.1, and 589.6 eV. They could be deconvoluted into Cr(III) peaks of Cr 2p_3/2_ (577.5 eV) and Cr 2p_1/2_ (586.9 and 588.1 eV), corresponding to the characteristics of Cr_2_O_3_ [72,73]. The 2 peaks at 578.8 and 589.6 eV could be deconvoluted into Cr(VI) peaks of Cr 2p_3/2_ and Cr 2p_1/2_ (Fig. 3F) [74]. The core-level spectrum of C 1s was deconvoluted into a main peak at 284.8 eV and a secondary peak at 285.9 eV, corresponding to C–C and C–OH bonds [75,76], respectively (Fig. 3G). The O 1s core-level spectrum was deconvoluted into 3 peaks at 531.7 eV (–OH/adsorbed oxygen), 533.0 eV (C–O), and 533.9 eV (adsorbed water) (Fig. 3H) [73,77,78]. The S 2p core-level spectrum was deconvoluted into 3 peaks at 168.2, 169.4, and 170.1 eV, attributed to S 2P_3/2_ and S 2P_1/2_ states in sulfonate groups, indicating the presence of SDBS molecules and/or DBS^−^ groups within the Au–Ag–Cr NCs (Fig. 3I) [79,80]. Collectively, these characterization results confirm that the synthesized ternary metal nanomaterials are primarily Au–Ag–Cr NCs.

### Selective and quantitative detection of Hg^2+^ and ClO^−^ using Au–Ag–Cr NCs and characterization of their mixtures

**Fig. 4.**
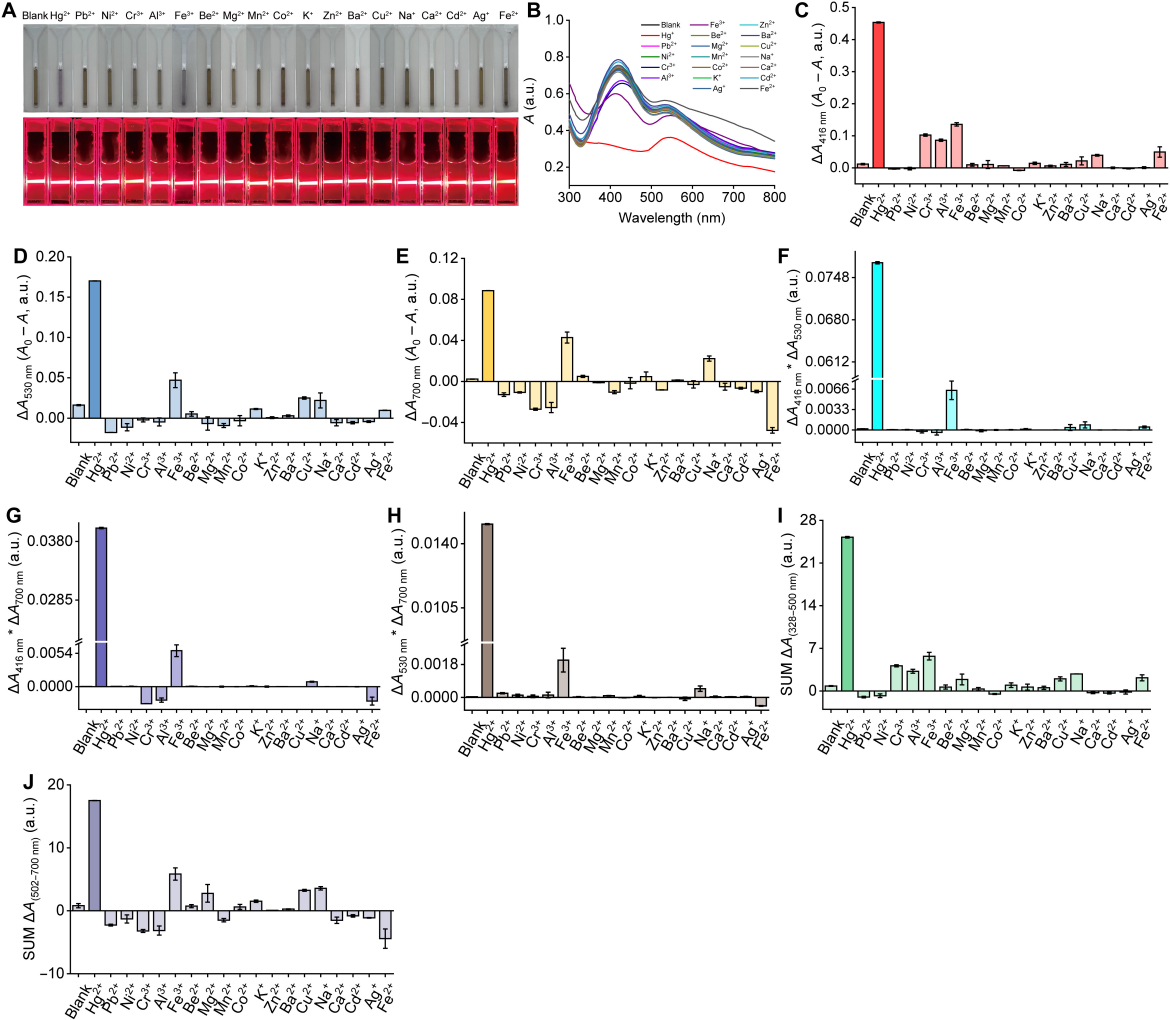
The selectivity of Au–Ag–Cr NCs for Hg^2+^. (A and B) Photographs showing changes in the color and Tyndall effect (A) of the Au–Ag–Cr NC solution mixed with different metal ions and the corresponding changes in absorption spectra (B). (C to E) Changes in the single channels after mixing Au–Ag–Cr NCs with different metal ions, including ∆*A*_416 nm_ (C), ∆*A*_530 nm_ (D), and ∆*A*_700 nm_ (E). (F to J) Changes in the combination channels after mixing Au–Ag–Cr NCs with different metal ions [including ∆*A*_416 nm_ × ∆*A*_530 nm_, ∆*A*_416 nm_ × ∆*A*_700 nm_, ∆*A*_530 nm_ × ∆*A*_700 nm_, SUM Δ*A*_(328–500 nm)_, and SUM Δ*A*_(502–700 nm)_]. *A*_0_ and *A* represent the absorbance without and with the analyte, respectively. Au–Ag–Cr NCs was prepared at a concentration ratio of 0.25:1.75:0.5 mM and diluted 1.33-fold. All metal ion concentration, 100 μM.

To detect the selectivity of Au–Ag–Cr NCs toward specific analytes, Au–Ag–Cr NCs were reacted with the 2 kinds of target substances, including (a) 19 metal ions: Hg^2+^, Pb^2+^, Ni^2+^, Cr^3+^, Al^3+^, Fe^3+^, Be^2+^, Mg^2+^, Mn^2+^, Co^2+^, K^+^, Zn^2+^, Ba^2+^, Cu^2+^, Na^+^, Ca^2+^, Cd^2+^, Ag^+^, and Fe^2+^ and (b) 13 anions/reducing agents: ClO^−^, Cl^−^, H_2_O_2_, I^−^, AC^−^, PO_4_^3−^, HCO_3_^−^, H_2_PO_4_^−^, OH^−^, DA, AA, Br^−^, and ClO_4_^−^ (all at a concentration of 100 μM). After 5 min of reaction at room temperature, we recorded the color, Tyndall effect, and UV-visible absorption spectrum of their mixed solutions. As shown in Fig. 4A, among the 19 metal ions tested, only Hg^2+^ induced a distinct color change in the Au–Ag–Cr NC solution, shifting from light brown to light purple (Fig. 4A), this change was accompanied by a significant decrease in absorption at 416 and 530 nm (Fig. 4B). In contrast, other metal ions exhibited negligible selectivity in terms of solution color, Tyndall effect, or absorption spectra compared with the blank control, except that Fe^3+^ caused a slight color change and a slight reduction in the absorption band at 416 nm (Fig. 4A and B; note that the interference of Fe^3+^ can be excluded using the characteristic absorption peak changes in the absorption spectra and the selected combined signal channels described in the next paragraph).

Initially, we selected 3 specific wavelengths (Δ*A*_416 nm_, Δ*A*_530 nm_, and Δ*A*_700 nm_) to compare the responses of Hg^2+^ against interfering metal ions. Figure 4C to E demonstrated that Au–Ag–Cr NCs showed the most significant absorption response to Hg^2+^ across these 3 channels [Δ*A*_416 nm_ = 0.454 absorbance units (a.u.), Δ*A*_530 nm_ = 0.170 a.u., and Δ*A*_700 nm_ = 0.088 a.u.], and these values were at least 3.34, 3.62, and −1.85 times (these values were calculated compared to Fe^3+^ with the second highest responses) higher than those of other interfering metal ions, respectively (due to the interference response being greater than zero). To reveal the hidden features in the response changes, enhance the signal-to-noise ratio between the target analyte and interferences, and improve detection sensitivity, we opted to combine the absorptions at different wavelengths for an integrated analysis. Through joint channel analysis, we found that combining channels could significantly enhance the selectivity and sensitivity for detecting Hg^2+^ compared to single-channel detection. As depicted in Fig. 4F to H, merging the absorption changes at 2 fixed wavelengths yielded Δ*A*_416 nm_ × Δ*A*_530 nm_, Δ*A*_416 nm_ × Δ*A*_700 nm_, and Δ*A*_530 nm_ × Δ*A*_700 nm_. The changes by Hg^2+^ at these 3 combining channels were 0.077, 0.040, and 0.015 a.u., respectively, which were at least 12.03, 6.90, and 7.50 times higher than that of other metal ions. In addition, the absorption changes across different wavelength ranges were summed to obtain SUM Δ*A*_(328–500 nm)_ and SUM Δ*A*_(502–700 nm)_. The changes by Hg^2+^ at these 2 combining channels were 25.23 and 17.51 a.u., respectively, which were at least 4.41 and 3.00 times higher than those for other metal ions (Fig. 4I and J). These results indicated that combining 2 channels can significantly enhance the selectivity for Hg^2+^. As shown in Table 1, under a single channel (such as Δ*A*_530 nm_), the response ratio for Hg^2+^ and interfering metal ions could reach at least 3.62 times, whereas, under the combination channels (such as Δ*A*_416 nm_ × Δ*A*_530 nm_), the response ratio could reach at least 12.03 times. On the basis of the 8 different readout signal modes of Au–Ag–Cr NCs (single signal channel and double signal combination channel), Hg^2+^ could be detected rapidly (approximately 2 min; Fig. S12A to C) and selectively (Fig. 4C to J). Since Au–Ag–Cr NCs were most sensitive to Hg^2+^ among the metal ions, the anti-interference capability of Au–Ag–Cr NCs in the presence of other metal ions or anions/reducing agents was also investigated. When Hg^2+^ coexists with various metal ions and anions/reducing agents (Fig. S13A and B), the color, Tyndall effect, and absorption responses of the Au–Ag–Cr NC solution at 2 wavelengths were similar to those observed with Hg^2+^ alone (differing by only 0.0003 to 0.007 a.u.). This result indicates that the Au–Ag–Cr NCs maintain excellent anti-interference capabilities in the presence of other metal ions or anions/reducing agents, which do not interfere with the detection of Hg^2+^ by the Au–Ag–Cr NCs.

**Table 1.** Comparison of selectivity and LODs for Hg^2+^ in different signal channels (including single channels and the combined channels)

Channel	Selectivity of Hg^2+^	LOD
Δ*A*_416 nm_	0.454 a.u.	0.039 μM
	3.34 times	
Δ*A*_530 nm_	0.17 a.u.	0.203 μM
	3.62 times	
Δ*A*_700 nm_	0.088 a.u.	0.167 μM
	−1.85 times	
Δ*A*_416 nm_ × Δ*A*_530 nm_	0.077 a.u.	0.027 nM
	12.03 times	
Δ*A*_416 nm_ × Δ*A*_700 nm_	0.040 a.u.	0.018 nM
	6.90 times	
Δ*A*_530 nm_ × Δ*A*_700 nm_	0.0015 a.u.	0. 183 nM
	7.50 times	
SUM Δ*A*_(328–500 nm)_	25.23 a.u.	0.646 μM
	4.41 times	
SUM Δ*A*_(502–700 nm)_	17.51 a.u.	2.16 μM
	3.00 times	

**Fig. 5.**
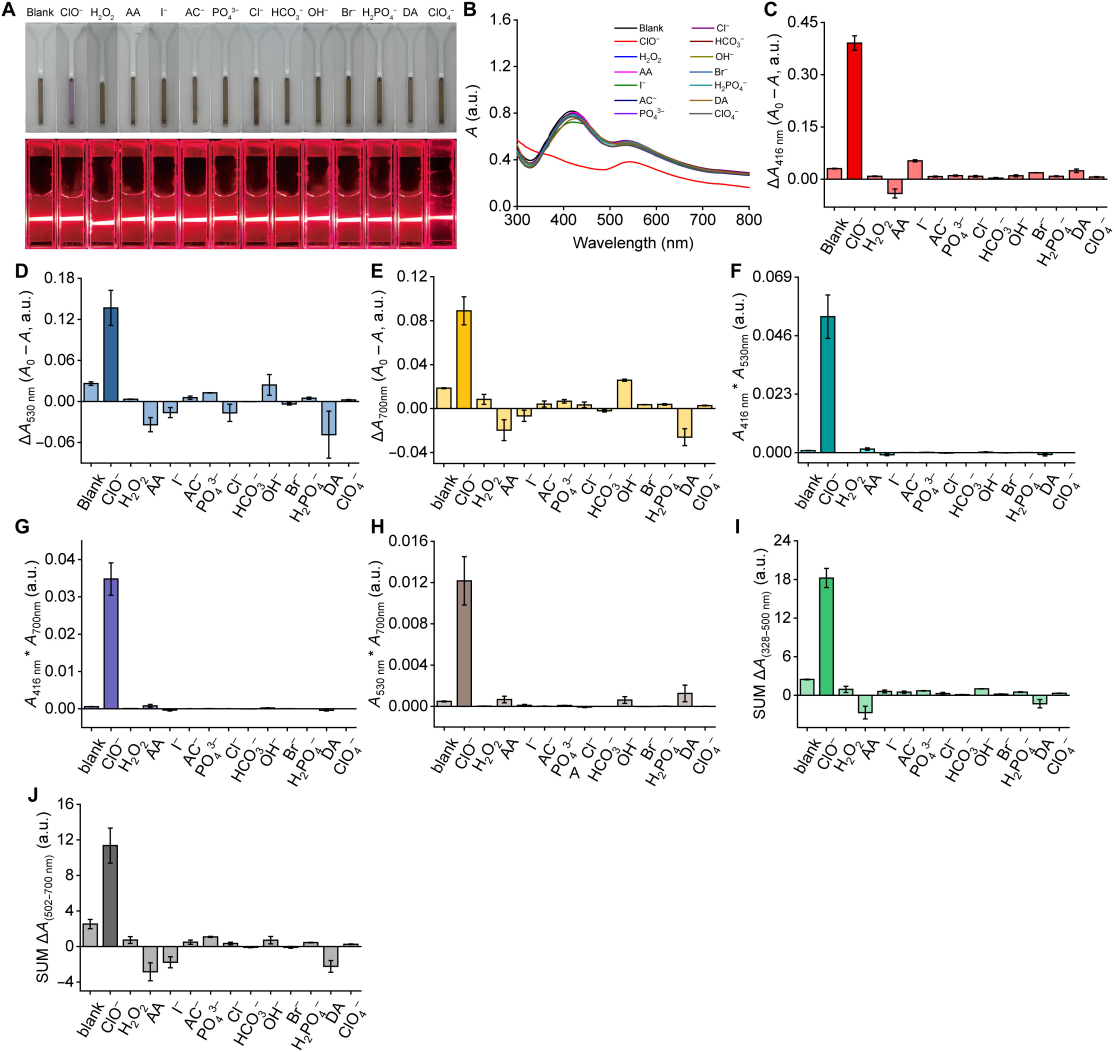
Selectivity of Au–Ag–Cr NCs toward ClO^−^. (A and B) Changes in the color and Tyndall effect (A) of the mixed solution of anions or reductants with Au–Ag–Cr NCs and the corresponding absorption spectrum changes (B). (C to E) Changes in the single signal channels after mixing Au–Ag–Cr NCs with different substances, including ∆*A*_416 nm_ (C), ∆*A*_530 nm_ (D), and ∆*A*_700 nm_ (E). (F to J) Changes in the combined channels after mixing Au–Ag–Cr NCs with different substances [including ∆*A*_416 nm_ × ∆*A*_530 nm_, ∆*A*_416 nm_ × ∆*A*_700 nm_, ∆*A*_530 nm_ × ∆*A*_700 nm_, SUM Δ*A*_(328–500 nm)_, and SUM Δ*A*_(502–700 nm)_]. *A*_0_ and *A* represent the absorbance in the absence and presence of analyte, respectively. Au–Ag–Cr NCs was prepared at a concentration ratio of 0.25:1.75:0.5 mM and diluted 1.33 times. All anions/reductants, 100 μM.

When adding another kind of analyte (13 anions/reductants) to Au–Ag–Cr NCs, the response to ClO^−^ was the most pronounced. In the presence of ClO^−^, the color of the Au–Ag–Cr NC solution changed from light brown to light purple (Fig. 5A), accompanied by a sharp decrease in the characteristic absorption bands of Ag NPs and Au NPs (Fig. 5B), whereas no significant selective characteristics were observed for other interfering substances. For ClO^−^, we also selected 3 discrete channels (Δ*A*_416 nm_, Δ*A*_530 nm_, and Δ*A*_700 nm_). Figure 5C to E showed that the Au–Ag–Cr NCs exhibited the most pronounced absorption response to ClO^−^ across 3 discrete channels (Δ*A*_416 nm_ = 0.391 a.u., Δ*A*_530 nm_ = 0.137 a.u., and Δ*A*_700 nm_ = 0.89 a.u.), which were at least 7.40, 2.8, and −3.42 times higher than that of other interfering substances, respectively. By combining the changes in absorption at 2 fixed wavelengths, Δ*A*_416 nm_ × Δ*A*_530 nm_, Δ*A*_416 nm_ × Δ*A*_700 nm_, and Δ*A*_530 nm_ × Δ*A*_700 nm_ for ClO^−^ were 0.0535, 0.0348, and 0.0122 a.u., respectively, which were at least 38.49, 43.37, and 9.67 times greater than that of other interfering substances (Fig. 5F to H). By summing the absorption changes across different wavelength ranges, the SUM Δ*A*_(328–500 nm)_ and SUM Δ*A*_(502–700 nm)_ for ClO^−^ were 18.22 and 11.35 a.u., respectively, which were at least −6.77 and 3.98 times higher than those of other interfering substances (Fig. 5I and J). The results demonstrated that the selectivity and sensitivity toward ClO^−^ could also be improved through combined channel analysis. As shown in Table 2, on the basis of the 8 different readout signal patterns described above, ClO^−^ could be detected rapidly (approximately 2 min; Fig. S12D to F) and selectively (Fig. 5C to J). Furthermore, when ClO^−^ coexisted with various metal ions and anions/reductants (Fig. S13C and D), the color, Tyndall effect, and the absorption responses of the Au–Ag–Cr NC solution at 2 wavelengths were similar to those observed when ClO^−^ was present alone (differing by only 0.0001 to 0.008 a.u.). These results indicate that the presence of metal ions or anions/reductants does not interfere with the measurement of ClO^−^ by Au–Ag–Cr NCs, which maintained good anti-interference capabilities.

**Table 2.** Comparison of selectivity and LODs for ClO^−^ in different signal channels (including single channels and 2-signal combination channels)

Channel	Selectivity of ClO^−^	LOD
Δ*A*_416 nm_	0.391 a.u.	0.043 μM
	7.40 times	
Δ*A*_530 nm_	0.137 a.u.	0.449 μM
	2.83 times	
Δ*A*_700 nm_	0.89 a.u.	0.200 μM
	−3.42 times	
Δ*A*_416 nm_ × Δ*A*_530 nm_	0.0535 a.u.	0.043 nM
	38.49 times	
Δ*A*_416 nm_ × Δ*A*_700 nm_	0.0348 a.u.	0.026 nM
	43.37 times	
Δ*A*_530 nm_ × Δ*A*_700 nm_	0.0122 a.u.	0.196 nM
	9.67 times	
SUM Δ*A*_(328–500 nm)_	18.22 a.u.	2.17 μM
	−6.77 times	
SUM Δ*A*_(502–700 nm)_	11.35 a.u.	3.65 μM
	3.98 times	

**Fig. 6.**
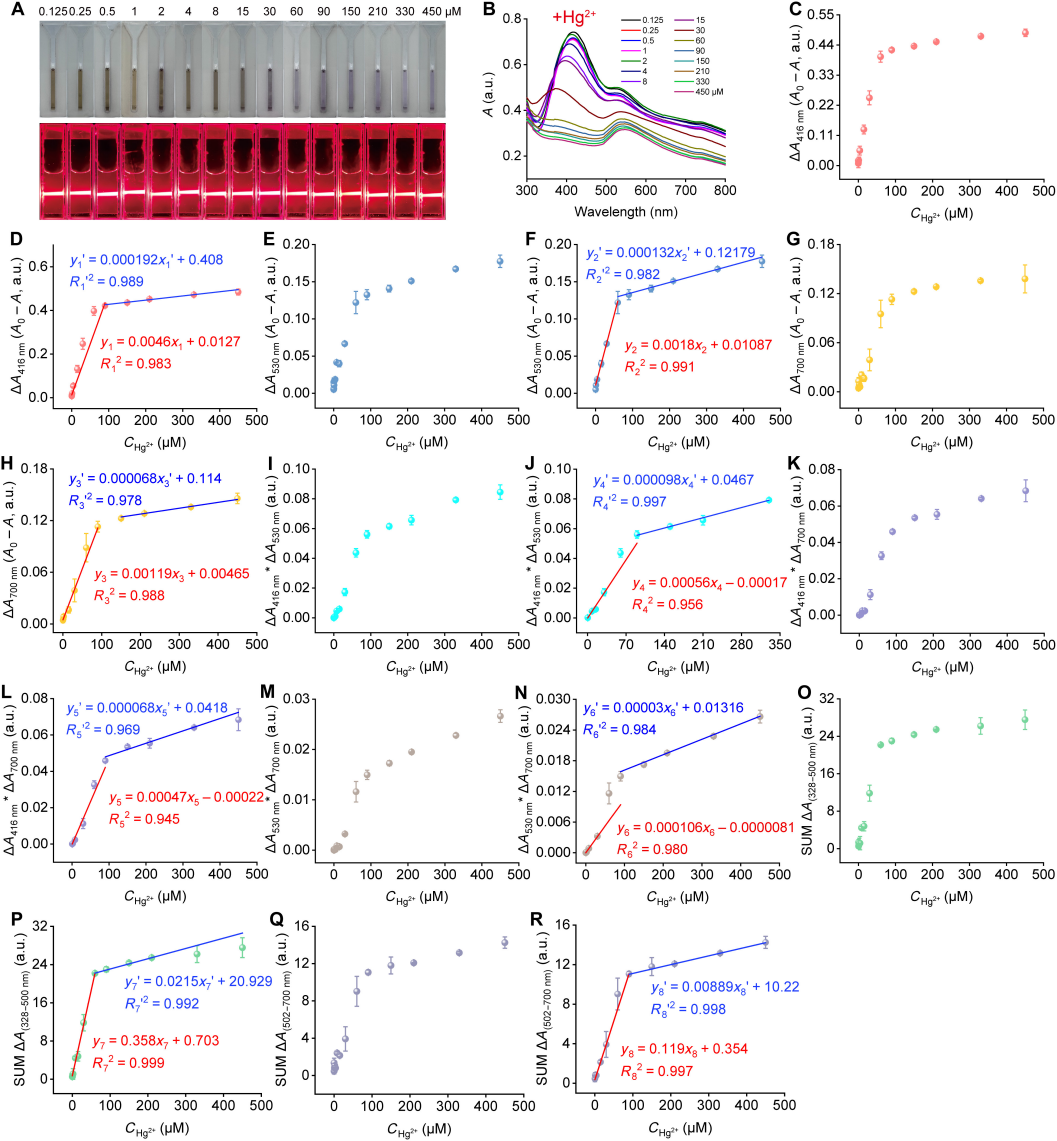
A multimodal colorimetric method based on Au–Ag–Cr NCs for detecting Hg^2+^. (A and B) Photographs of the color and Tyndall effect (A) of Au–Ag–Cr NCs (0.25:1.75:0.5 mM, diluted 1.33 times) after adding different concentrations of Hg^2+^ (0 to 450 μM) and the corresponding changes in absorption spectra (B). Note that each absorption spectrum was recorded after 5 min of the reaction. (C to H) The single-channel responses of Au–Ag–Cr NCs after adding different concentrations of Hg^2+^ [including absorption changes at 416 (C), 530 (E), and 700 nm (G)] and their corresponding linear calibration curves (D, F, and H). (I to N) The combined channel responses of Au–Ag–Cr NCs after adding different concentrations of Hg^2+^ [including Δ*A*_416 nm_ × Δ*A*_530 nm_ (I), Δ*A*_416 nm_ × Δ*A*_700 nm_ (K), Δ*A*_530 nm_ × Δ*A*_700 nm_ (M), SUM Δ*A*_(328–500 nm)_ (O), and SUM Δ*A*_(502–700 nm)_ (Q)] and their corresponding calibration curves (J, L, N, P, and R).

Given the superior sensitivity of Au–Ag–Cr NCs toward Hg^2+^ and ClO^−^ compared to other analytes, we further explored the colorimetric response of Au–Ag–Cr NCs to varying concentrations of Hg^2+^ and ClO^−^. In the range of 0.125 to 30 μM for Hg^2+^, there was little change in the solution color, while the characteristic absorption peaks of Ag NPs and Au NPs gradually decreased, along with visible shift of the characteristic peak of Ag NPs (Fig. 6A and B). As the concentration of Hg^2+^ increased to 30 μM, the Au–Ag–Cr NCs underwent a color change from light brown to light purple with a significant decrease in absorption. However, starting at 60 μM, the decrease in absorption became more gradual, showing minimal change until around 450 μM. For the single channels, Δ*A*_416 nm_, Δ*A*_530 nm_, and Δ*A*_700 nm_ exhibited a positive correlation within a certain concentration range (Fig. 6C, E, and G); the changes in absorption of Au–Ag–Cr NCs exhibited linear relationships with the variations of Hg^2+^ concentration in the ranges of 0.125 to 90 μM (Δ*A*_416 nm_) and 90 to 450 μM (Δ*A*_416 nm_), 0.125 to 60 μM (Δ*A*_530 nm_) and 60 to 450 μM (Δ*A*_530 nm_), as well as 0.125 to 90 μM (Δ*A*_700 nm_) and 150 to 450 μM (Δ*A*_700 nm_). Their calibration equations were *y*_1_ = 0.0046*x*_1_ + 0.0127 (*R*_1_^2^ = 0.983) and *y*_1_′ = 0.000192*x*_1_′ + 0.408 (*R*_1_′^2^ = 0.989; Fig. 6D); *y*_2_ = 0.0018*x*_2_ + 0.01087 (*R*_2_^2^ = 0.991) and *y*_2_′ = 0.000132*x*_2_′ + 0.12179 (*R*_2_′^2^ = 0.982; Fig. 6F); as well as *y*_3_ = 0.00119*x*_3_ + 0.00465 (*R*_3_^2^ = 0.988) and *y*_3_′ = 0.000068*x*_3_′ + 0.114 (*R*_3_′^2^ = 0.978; Fig. 6H). According to the 3σ principle, their limits of detection (LODs) for Hg^2+^ were 0.039 μM (Δ*A*_416 nm_), 0.203 μM (Δ*A*_530 nm_), and 0.167 μM (Δ*A*_700 nm_), respectively.

Furthermore, on the basis of the responsiveness to Hg^2+^, the corresponding combined channels Δ*A*_416 nm_ × Δ*A*_530 nm_, Δ*A*_416 nm_ × Δ*A*_700 nm_, Δ*A*_530 nm_ × Δ*A*_700 nm_, SUM Δ*A*_(328–500 nm)_, and SUM Δ*A*_(502–700 nm)_ exhibited a positive correlation within a certain concentration range (Fig. 6I, K, M, O, and Q). These combined channels presented linear relationships with Hg^2+^ in the ranges of 0.25 to 90 μM (Δ*A*_416 nm_ × Δ*A*_530 nm_) and 90 to 330 μM (Δ*A*_416 nm_ × Δ*A*_530 nm_), 0.5 to 90 μM (Δ*A*_416 nm_ × Δ*A*_700 nm_) and 90 to 450 μM (Δ*A*_416 nm_ × Δ*A*_700 nm_), 0.125 to 60 μM (Δ*A*_530 nm_ × Δ*A*_700 nm_) and 90 to 450 μM (Δ*A*_530 nm_ × Δ*A*_700 nm_), 0.125 to 60 μM [SUM Δ*A*_(328–500 nm)_] and 60 to 450 μM [SUM Δ*A*_(328–500 nm)_], and 0.125 to 90 μM [SUM Δ*A*_(502–700 nm)_] and 90 to 450 μM [SUM Δ*A*_(502–700 nm)_]. Their calibration equations were as follows: *y*_4_ = 0.00056*x*_4_ − 0.00017 (*R*_4_^2^ = 0.956) and *y*_4_′ = 0.000098*x*_4_′ + 0.0467 (*R*_4_′^2^ = 0.997, Δ*A*_416 nm_ × Δ*A*_530 nm_, LOD = 0.027 nM; Fig. 6J), *y*_5_ = 0.00047*x*_5_ – 0.00022 (*R*_5_^2^ = 0.945) and *y*_5_′ = 0.000068*x*_5_′ + 0.0418 (*R*_5_′^2^ = 0.99, Δ*A*_416 nm_ × Δ*A*_700 nm_, LOD = 0.018 nM; Fig. 6L), *y*_6_ = 0.000106*x*_6_ – 0.0000081 (*R*_6_^2^ = 0.980) and *y*_6_′ = 0.00003*x*_6_′ + 0.01316 (*R*_6_′^2^ = 0.984, Δ*A*_530 nm_ × Δ*A*_700 nm_, LOD = 0.183 nM; Fig. 6N), *y*_7_ = 0.0358*x*_7_ + 0.703 (*R*_7_^2^ = 0.999) and *y*_7_′ = 0.0215*x*_7_′ + 20.929 [*R*_7_′^2^ = 0.992, SUM Δ*A*_(328–500 nm)_, LOD = 0.646 μM; Fig. 6P], and *y*_8_ = 0.119*x*_8_ + 0.354 (*R*_8_^2^ = 0.997) and *y*_8_′ = 0.00889*x*_8_′ + 10.22 [*R*_8_′^2^ = 0.998, SUM Δ*A*_(502–700 nm)_, LOD = 2.16 μM; Fig. 6R]. Among the above channels, the 2 combined channels of Δ*A*_416 nm_ × Δ*A*_700 nm_ and Δ*A*_416 nm_ × Δ*A*_530 nm_ exhibited the highest detection sensitivity for Hg^2+^ detection, with LODs of 0.018 and 0.027 nM, respectively. These LOD values are below the maximum allowable level (10 nM) for inorganic Hg^2+^ in drinking water, as stipulated by regulatory standards. Compared to methods reported in other studies, the approaches used here offer a comparable linear range and even lower LODs for Hg^2+^ (Table S1). Through the combined channels, the high sensitivity and selectivity of detecting Hg^2+^ can be achieved.

**Fig. 7.**
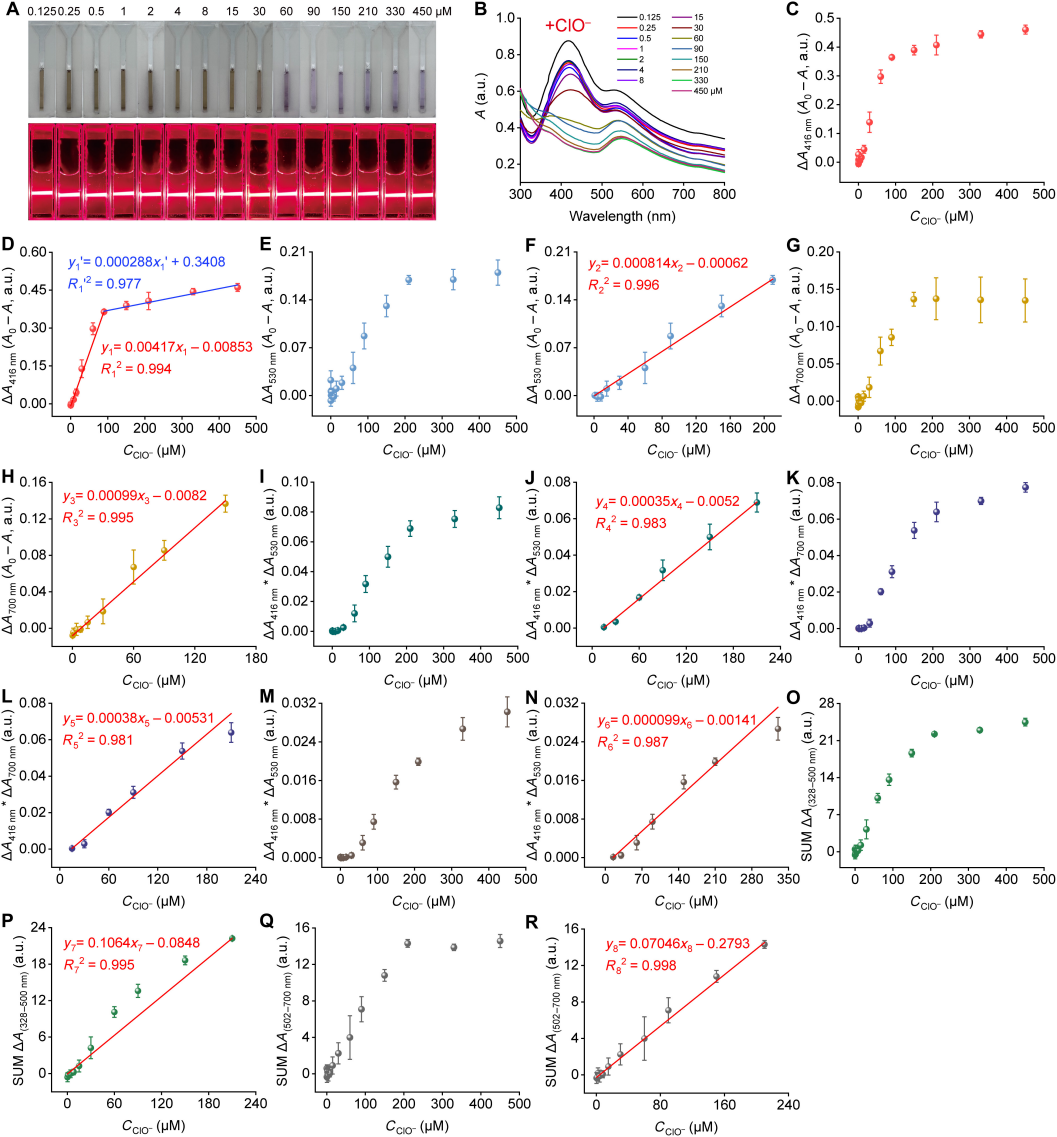
Detection of ClO^−^ based on Au–Ag–Cr NCs using a multimodal colorimetric method. (A and B) Photographs showing color and Tyndall effect (A) of Au–Ag–Cr NCs (0.25:1.75:0.5 mM, diluted 1.33 times) after the addition of different concentrations of ClO^−^ (0 to 450 μM) and the corresponding changes in absorption spectra (B). Note that each absorption spectrum was measured after 5 min of the reaction. (C to J) Single-channel responses of Au–Ag–Cr NCs after the addition of different concentrations of ClO^−^, showing absorption changes at 416 (C), 530 (E), and 700 nm (G) along with their corresponding linear calibration curves (D, F, and H). (I to R) The combined channel responses of Au–Ag–Cr NCs after the addition of different concentrations of ClO^−^, including Δ*A*_416 nm_ × Δ*A*_530 nm_ (I), Δ*A*_416 nm_ × Δ*A*_700 nm_ (K), Δ*A*_530 nm_ × Δ*A*_700 nm_ (M), SUM Δ*A*_(328–500 nm)_ (O), and SUM Δ*A*_(502–700 nm)_ (Q), along with their corresponding calibration curves (J, L, N, P, and R).

Similarly, within the concentration range of ClO^−^ from 0.125 to 30 μM, there was little change in the color of the mixed solution as ClO^−^ concentration increased. When the concentration reached 60 μM, the color of the solution shifted from light brown to light purple, and no further color change occurred (Fig. 7A). The characteristic absorption peak of Ag NPs (around 416 nm) exhibited a significant decrease between 0.125 and 60 μM and completely disappeared at 60 μM, while the peak position displayed no notable shift. Meanwhile, the characteristic absorption peak of Au NPs (around 530 nm) gradually decreased within the ClO^−^ concentration range of 0.125 to 450 μM, with no obvious shift. For the single-channel response, the absorption changes of Au–Ag–Cr NCs were linearly related to ClO^−^ concentration in the ranges of 0.25 to 90 μM (Δ*A*_416 nm_), and 90 to 450 μM (Δ*A*_416 nm_), 1 to 210 μM (Δ*A*_530 nm_), and 0.25 to 150 μM (Δ*A*_700 nm_). Their corresponding calibration equations were *y*_1_ = 0.00417*x*_1_ − 0.00853 (*R*_1_^2^ = 0.994; Fig. 7D) and *y*_1_′ = 0.000288*x*_1_′ + 0.3408 (*R*_1_′^2^ = 0.977; Fig. 7D), *y*_2_ = 0.000814*x*_2_ − 0.00062 (*R*_2_^2^ = 0.996; Fig. 7F), and *y*_3_ = 0.00099*x*_3_ − 0.0082 (*R*_3_^2^ = 0.995; Fig. 7H). On the basis of the 3σ principle, their LODs for ClO^−^ were 0.043 μM (Δ*A*_416 nm_), 0.449 μM (Δ*A*_530 nm_), and 0.200 μM (Δ*A*_700 nm_).

Furthermore, on the basis of the responsiveness of ClO^−^, the corresponding composite channels Δ*A*_416 nm_ × Δ*A*_530 nm_, Δ*A*_416 nm_ × Δ*A*_700 nm_, Δ*A*_530 nm_ × Δ*A*_700 nm_, SUM Δ*A*_(328–500 nm)_, and SUM Δ*A*_(502–700 nm)_ exhibited a positive correlation within a certain concentration range (Fig. 7I, K, M, O, and Q). These composite channels had a linear relationship with ClO^−^ in the respective ranges: 15 to 210 μM (Δ*A*_416 nm_ × Δ*A*_530 nm_), 15 to ~210 μM (Δ*A*_416 nm_ × Δ*A*_700 nm_), 15 to 330 μM (Δ*A*_530 nm_ × Δ*A*_700 nm_), 0.25 to ~210 μM [SUM Δ*A*_(328–500 nm)_], and 0.125 to 210 μM [SUM Δ*A*_(502–700 nm)_]. Their corresponding calibration equations were *y*_4_ = 0.00035*x*_4_ − 0.0052 (*R*_4_^2^ = 0.983, Δ*A*_416 nm_ × Δ*A*_530 nm_, LOD of 0.043 nM; Fig. 7J), *y*_5_ = 0.00038*x*_5_ – 0.00531 (*R*_5_^2^ = 0.981, Δ*A*_416 nm_ × Δ*A*_700 nm_, LOD of 0.026 nM; Fig. 7L), *y*_6_ = 0.000099*x*_6_ – 0.00141 (*R*_6_^2^ = 0.987, Δ*A*_530 nm_ × Δ*A*_700 nm_, LOD of 0.196 nM; Fig. 7N), *y*_7_ = 0.1064*x*_7_ – 0.0848 (*R*_7_^2^ = 0.995, SUM Δ*A*_(328-500 nm)_, LOD of 2.17 μM; Fig. 7P), and *y*_8_ = 0.07046*x*_8_ – 0.2793 (*R*_8_^2^ = 0.998, SUM Δ*A*_(502-700 nm)_, LOD of 3.65 μM; Fig. 7R). Among these, the combined Δ*A*_416 nm_ × Δ*A*_700 nm_ and Δ*A*_416 nm_ × Δ*A*_530 nm_ combination channels had the highest sensitivity with LODs of 0.026 and 0. 043 nM, respectively, which were below the minimum allowable level (0.2 mg/l, ~388.7 nM, calculated as free chlorine) of ClO^−^ in drinking water permitted by the World Health Organization. Compared to other previously reported ClO^−^ detection methods, our proposed method has a comparable linear range and even lower LOD (Table S2). The results show that Hg^2+^ or ClO^−^ can be sensitively and selectively detected using dual signal combination channels (Δ*A*_416 nm_ × Δ*A*_700 nm_ and Δ*A*_416 nm_ × Δ*A*_530 nm_), respectively.

**Fig. 8.**
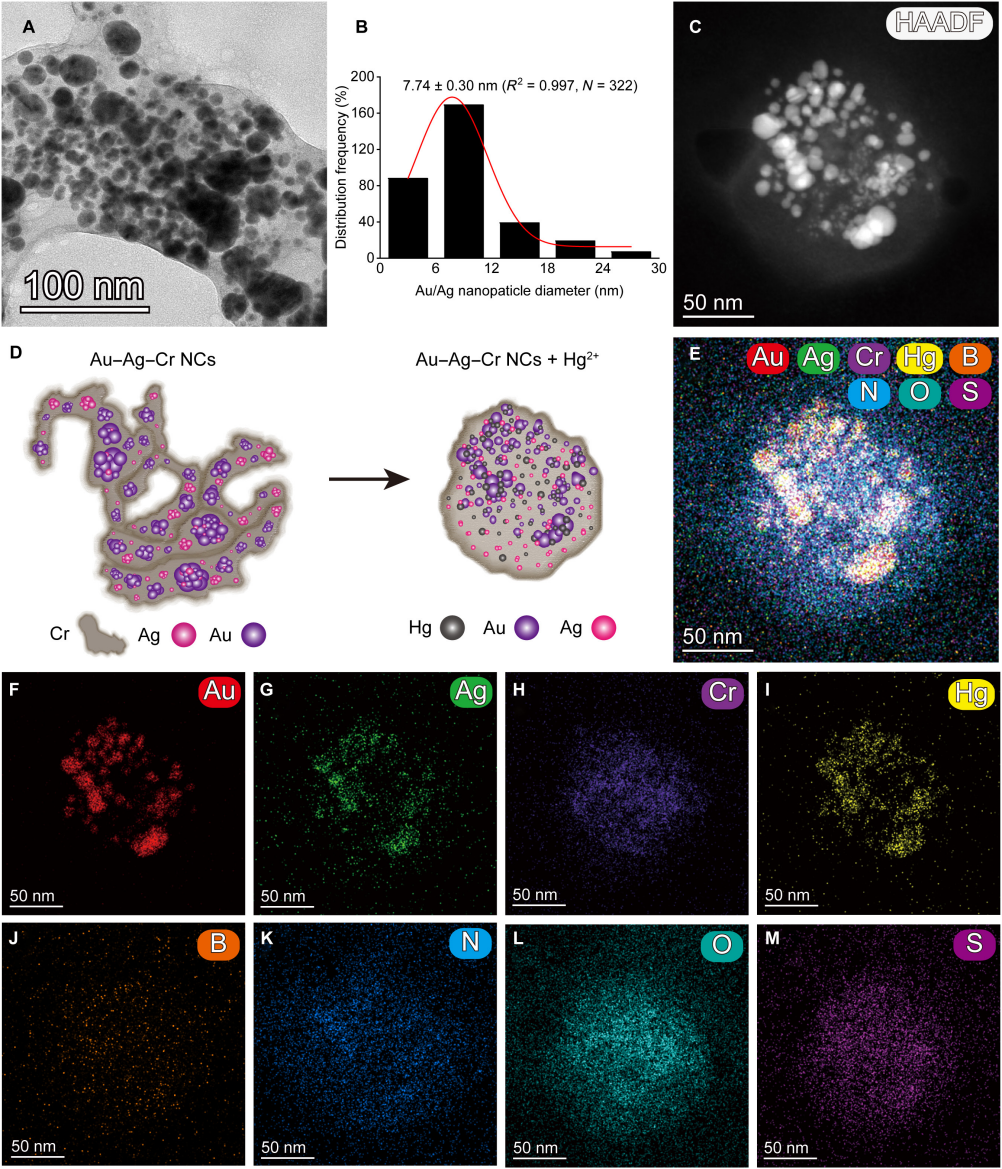
Characterization of Au–Ag–Cr NCs and Hg^2+^ mixture. (A, C, and E to M) TEM (A), HAADF-STEM (C) images, and EDS elemental mapping (E to M) of Au–Ag–Cr NCs and Hg^2+^ mixture. (B) Statistical histogram of spherical Au/Ag NPs diameter in the mixture obtained by processing TEM images with ImageJ software. (D) Schematic representation of the state change upon the addition of Hg^2+^ into Au–Ag–Cr NCs. Scale bars, 100 and 50 nm.

**Fig. 9.**
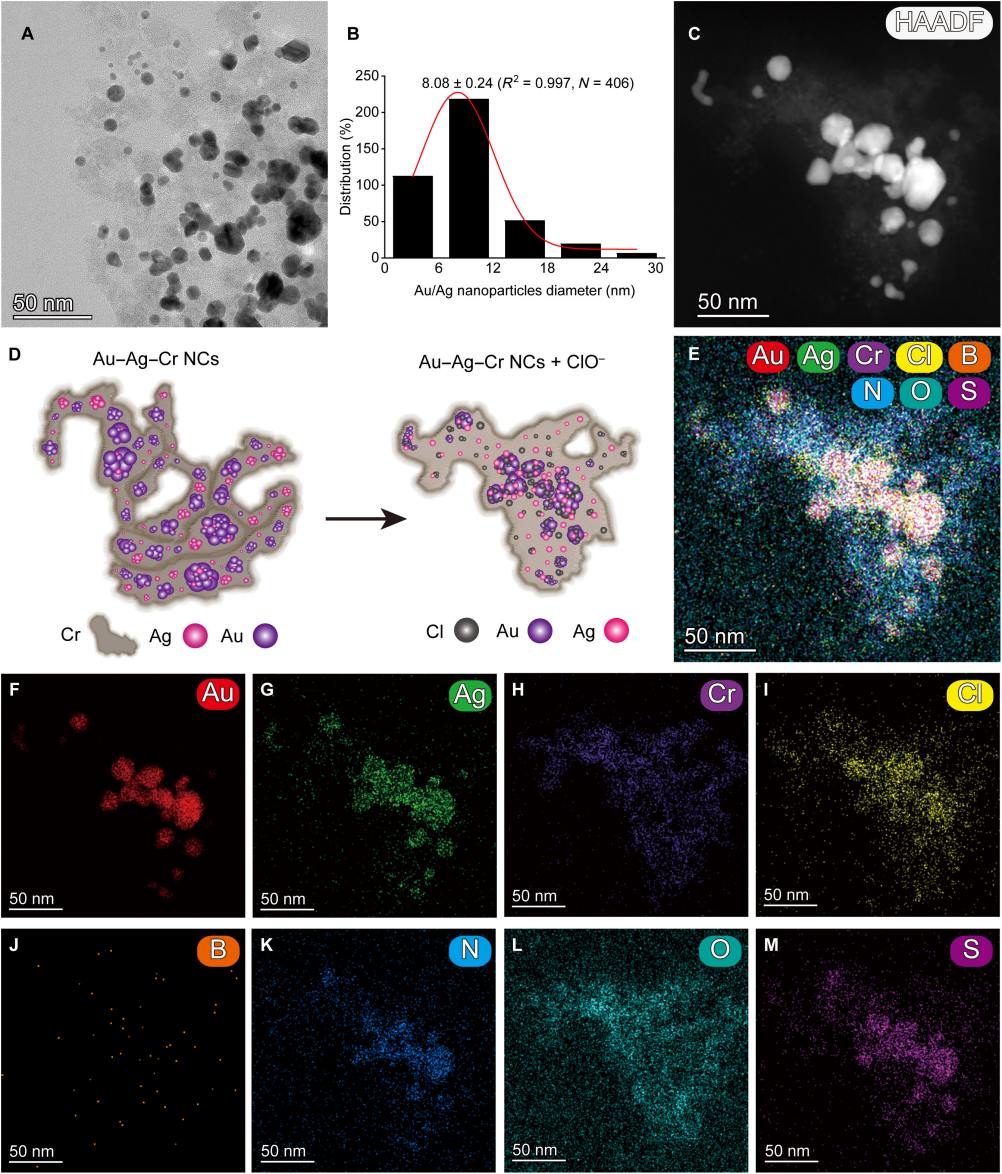
Characterization of Au–Ag–Cr NCs and ClO^−^ mixture. (A, C, and E to M) TEM (A), HAADF-STEM (C) images, and EDS elemental mapping (E to M) of Au–Ag–Cr NCs and ClO^−^ mixture. (B) Statistical histogram of spherical Au–Ag NPs diameter in the mixture obtained by processing TEM images with ImageJ software. (D) Schematic representation of the state change upon the addition of ClO^−^ into Au–Ag–Cr NCs. Scale bars, 50 nm.

To examine the impact of Hg^2+^ on the morphology of Au–Ag–Cr NCs, we performed characterization using TEM. Figure 8A displayed an uneven size distribution, containing both small particles and agglomerated oversized particles of Au–Ag–Cr NCs. Simultaneously, the particle diameter distribution decreased from 14.15 ± 0.63 to 7.74 ± 0.30 nm (*R*^2^ = 0.997, *N* = 322; Fig. 8B), an effect attributed to the etching effect of Hg^2+^ on Au/Ag NPs in Au–Ag–Cr NCs [23,81]. In addition, upon the addition of Hg^2+^, the edges of the Cr NBs became rounded, and branching was no longer observed (Fig. 8A, C, and D). EDS elemental mapping results (Fig. 8E to M) indicated that Hg, Au, and Ag in the mixture had a strong colocalization and the purple intensity of Cr NBs was reduced, while the N, O and S elements were uniformly distributed within the range of the Cr NBs. These results suggested that the etching effect of Hg^2+^ on Au/Ag NPs in Au–Ag–Cr NCs caused the Au/Ag NPs to shrink in size. This reduction led to a decrease in absorption intensity and blue shift of Ag NPs’ absorption peak (Fig. 8D) [82]. For the mixture of Au–Ag–Cr NCs and ClO^−^, we also conducted TEM characterization. Upon the addition of ClO^−^, the number of NPs on the Cr NBs was significantly reduced (Fig. 9A) and the average particle diameter decreased from 14.15 ± 0.63 to 8.08 ± 0.24 nm (*R*^2^ = 0.997, *N* = 406; Fig. 9B). EDS elemental mapping results further showed that Cl was distributed in the whole region of Au–Ag–Cr NCs and had strong colocalization with Au, Ag, and Cr, while the distribution of the other elements was less pronounced than the changes before the addition of ClO^−^ (Fig. 9C to M). These results indicated that exposure to ClO^−^ led to etching of Ag atoms from Au–Ag–Cr NCs, resulting in a reduction in Ag content and a subsequent decrease in absorption intensity (Fig. 9D) [24,83,84].

In addition, we further assessed the feasibility of the prepared Au–Ag–Cr NCs for detecting Hg^2+^ and ClO^−^ in real water samples (pond water and tap water; Tables 3 to 6 and Fig. S14). The recovery rates for spiked Hg^2+^ in pond water ranged from 101.29% to 132.72%, with relative standard deviations (RSDs) varying between 0.62% and 8.64%. The recovery rates for spiked Hg^2+^ in tap water samples ranged from 94.26% to 121.70%, with RSDs varying between 1.24% and 8.29% (Tables 3 and 4 and Fig. S14A and B). At different ClO^−^ spiking levels (15 to 210 μM; Tables 5 and 6 and Fig. S14C and D), the recovery rates for spiked ClO^−^ in pond water samples ranged from 100.95% to 117.93%, with RSDs varying between 0.46% and 3.46% [note that when the spiked concentration of ClO^−^ is 0 μM, the detected concentration under the channels SUM Δ*A*_(328–500 nm)_ and SUM Δ*A*_(502–700 nm)_ was relatively high because of the large amount of impurities present in the pond water], while the recovery rates for spiked ClO^−^ in tap water samples ranged from 96.11% to 120.00%, with RSDs varying between 1.68% and 4.69%.

**Table 3.** Detection of Hg^2+^ in pond water samples under 8 different channels

Analyte	Channel	Concentration of Hg^2+^ (μM)		Recovery	RSD
		Added	Detected	(%)	(%, *n* = 3)
Hg^2+^	Δ*A*_416 nm_	0	N.D.	–	–
		60	70.67	117.78	6.09
	Δ*A*_530 nm_	0	N.D.	–	–
		15	15.75	105.00	8.64
	Δ*A*_700 nm_	0	N.D.	–	–
		90	97.49	108.33	1.84
	Δ*A*_416 nm_ × Δ*A*_530 nm_	0	N.D.	–	–
		60	61.15	101.92	4.58
	Δ*A*_416 nm_ × Δ*A*_700 nm_	0	N.D.	–	–
		30	39.81	132.72	0.62
	Δ*A*_530 nm_ × Δ*A*_700 nm_	0	N.D.	–	–
		30	32.82	119.43	1.53
	SUM Δ*A*_(328–500 nm)_	0	N.D.	–	–
		30	30.38	101.29	2.73
	SUM Δ*A*_(502–700 nm)_	0	N.D.	–	–
		90	100.30	111.44	2.63

N.D., not detected.

**Table 4.** Detection of Hg^2+^ in tap water samples under 8 different channels

Analyte	Channel	Concentration of Hg^2+^ (μM)		Recovery	RSD
		Added	Detected	(%)	(%, *n* = 3)
Hg^2+^	Δ*A*_416 nm_	0	N.D.	–	–
		60	73.02	121.70	2.97
	Δ*A*_530 nm_	0	N.D.	–	–
		15	14.13	94.26	1.38
	Δ*A*_700 nm_	0	N.D.	–	–
		90	99.37	110.41	2.73
	Δ*A*_416 nm_ × Δ*A*_530 nm_	0	N.D.	–	–
		60	68.18	113.63	1.41
	Δ*A*_416 nm_ × Δ*A*_700 nm_	0	N.D.	–	–
		30	32.70	109.01	2.76
	Δ*A*_530 nm_ × Δ*A*_700 nm_	0	N.D.	–	–
		30	30.42	101.39	8.29
	SUM Δ*A*_(328–500 nm)_	0	N.D.	–	–
		30	30.69	102.30	4.65
	SUM Δ*A*_(502–700 nm)_	0	N.D.	–	–
		90	107.27	119.19	1.24

**Table 5.** Detection of ClO^−^ in pond water samples under 8 different channels

Analyte	Channel	Concentration of ClO^−^ (μM)		Recovery	RSD
		Added	Detected	(%)	(%, *n* = 3)
ClO^−^	Δ*A*_416 nm_	0	3.16	–	5.28
		60	70.76	117.93	2.94
	Δ*A*_530 nm_	0	5.16	–	2.22
		150	165.42	116.97	2.87
	Δ*A*_700 nm_	0	4.84	–	3.58
		150	186.41	103.56	0.46
	Δ*A*_416 nm_ × Δ*A*_530 nm_	0	3.37	–	7.50
		15	16.15	107.65	3.46
	Δ*A*_416 nm_ × Δ*A*_700 nm_	0	2.60	–	8.86
		15	16.19	107.92	0.72
	Δ*A*_530 nm_ × Δ*A*_700 nm_	0	4.95	–	5.80
		15	15.14	100.95	0.77
	SUM Δ*A*_(328–500 nm)_	0	8.2	–	5.39
		150	158.54	105.69	2.18
	SUM Δ*A*_(502–700 nm)_	0	6.75	–	2.88
		210	235.99	112.38	2.13

**Table 6.** Detection of ClO^−^ in tap water samples under 8 different channels

Analyte	Channel	Concentration of ClO^−^ (μM)		Recovery	RSD
		Added	Detected	(%)	(%, *n* = 3)
ClO^−^	Δ*A*_416 nm_	0	1.80	–	2.32
		60	61.39	102.31	4.32
	Δ*A*_530 nm_	0	7.74	–	6.21
		150	149.90	99.93	4.59
	Δ*A*_700 nm_	0	4.70	–	5.88
		150	180	120.00	1.68
	Δ*A*_416 nm_ × Δ*A*_530 nm_	0	1.54	–	2.05
		15	15.92	106.13	2.95
	Δ*A*_416 nm_ × Δ*A*_700 nm_	0	1.76	–	3.25
		15	14.69	97.97	3.04
	Δ*A*_530 nm_ × Δ*A*_700 nm_	0	1.82	–	5.92
		15	14.85	98.97	3.43
	SUM Δ*A*_(328–500 nm)_	0	0.76	–	0.49
		150	144.16	96.11	2.73
	SUM Δ*A*_(502–700 nm)_	0	2.11	–	2.17
		210	218.20	103.90	4.69

### Information processing and protection based on Au–Ag–Cr NCs

#### A multimodal colorimetric sensing system based on Au–Ag–Cr NCs for molecular logic computing

**Fig. 10.**
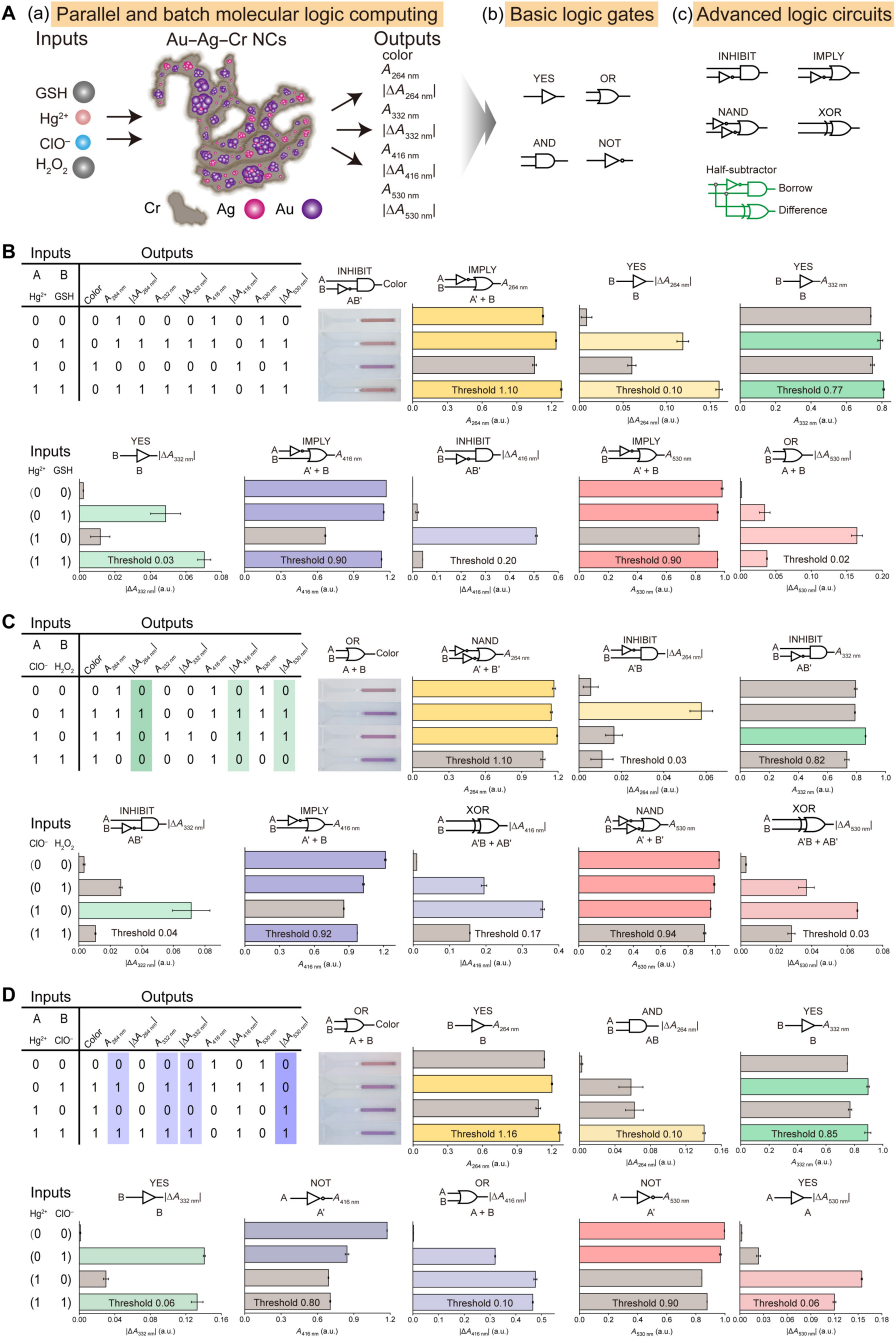
Parallel and batch molecular logic computing based on Au–Ag–Cr NCs. (A) Schematic illustration of the input–output relationships based on Au–Ag–Cr NCs and the constructed simple logic gates and complex logic circuits. (B to D) Multimode simple logic gates and complex cascaded circuits based on 9 signal outputs of Au–Ag–Cr NCs. The thresholds were respectively defined as follows: “purple”, *A*_264 nm_ > 1.10 (B), 1.10 (C), and 1.16 (D); |Δ*A*_264 nm_| > 0.10 (B), 0.03 (C), and 0.10 (D); *A*_332 nm_ > 0.77 (B), 0.82 (C), and 0.85 (D); |Δ*A*_332 nm_| > 0.03 (B), 0.04 (C), and 0.06 (D); *A*_416 nm_ > 0.90 (B), 0.92 (C), and 0.80 (D); |Δ*A*_416 nm_| > 0.20 (B), 0.17 (C), and 0.10 (D); *A*_530 nm_ > 0.90 (B), 0.94 (C), and 0.90 (D); |Δ*A*_530 nm_| > 0.02 (B), 0.03 (C), and 0.06 (D) are defined as “1”, otherwise as “0”. Au–Ag–Cr NCs, 0.25:1.75:0.50 mM, diluted 1.33 times. GSH and H_2_O_2_, 10 mM; Hg^2+^ and ClO^−^, 60 μM.

Leveraging the multisignal colorimetric responses of Au–Ag–Cr NCs to various substances, we constructed a series of basic logic gates and advanced cascaded logic circuits [Fig. 10A(b) and (c)]. Different substances added to Au–Ag–Cr NCs [such as Hg^2+^, ClO^−^, glutathione (GSH), or H_2_O_2_] were used as inputs, while the colorimetric changes in multisignal channels (absorbance, color, and their absolute differences) served as outputs [Fig. 10A(a)]. The presence and absence of substances are defined as logic “1” and logic “0”, respectively. The resulting purple color of the solution, absorbance, or their absolute differences (*A*_264 nm_ > 1.10, 1.10 or 1.16, |Δ*A*_264 nm_| > 0.10, 0.03 or 0.10, *A*_332 nm_ > 0.77, 0.82 or 0.85, |Δ*A*_332 nm_| > 0.03, 0.04 or 0.06, *A*_416 nm_ > 0.90, 0.92 or 0.80, |Δ*A*_416 nm_| > 0.20, 0.17 or 0.10, *A*_530 nm_ > 0.90, 0.94 or 0.90, |Δ*A*_530 nm_| > 0.02, 0.03 or 0.06) are defined as “1”, otherwise “0”. It is noted that the absorbance change at 264 nm is attributed to the signal response of Cr NBs in Au–Ag–Cr NCs to the additives. The selection of absorbance data at 332 nm and the different data processing methods for each channel are intended to increase the number of output channels. This allows for a great variety of logic calculations and provides enhanced storage capacity for subsequent information encoding.

When Hg^2+^ and GSH are introduced into Au–Ag–Cr NCs and different data processing methods are adopted, 4 distinct logic gates (YES, OR, INHIBIT, and IMPLY) can be constructed on the basis of the outputs from the 9 signal channels (Fig. 10B and Fig. S15A). The Au–Ag–Cr NCs (corresponding to input 0, 0) present a light brown solution, displaying 2 strong absorption bands at 416 and 530 nm (Fig. S15A). The presence of GSH alone (corresponding to input 0, 1) does not significantly alter the color of the solution but causes an increase in absorbance at 264 and 332 nm. Conversely, the presence of Hg^2+^ alone (corresponding to input 1, 0) induces the solution to appear purple while decreasing the absorbance at 264, 416, and 530 nm, with a slight increase at 332 nm. When both GSH and Hg^2+^ are added simultaneously to Au–Ag–Cr NCs (corresponding to input 1, 1), there is no significant change in the solution color, with absorbance only increasing at 264 and 332 nm (Fig. S15A). That is, when GSH or Hg^2+^ is present alone, they react with Cr NBs in Au–Ag–Cr NCs, leading to changes in the absorption values at 264 and 332 nm, while Hg^2+^ additionally causes a marked decrease in the characteristic absorption peaks of Au–Ag–Cr NCs; however, their combination does not change the solution color or the characteristic absorption peaks of the Au–Ag–Cr NCs. From the perspective of logical calculation to analyze the abovementioned input and output relationship, for the outputs of |Δ*A*_264 nm_|, A_332 nm_, and |Δ*A*_332 nm_|, the presence of GSH (logic input 1) results in a significant increase in these outputs, corresponding to the YES gate (Boolean algebra B). For the output of |Δ*A*_530 nm_|, the presence of either GSH or Hg^2+^ (logic input 1) results in |Δ*A*_530 nm_| exceeding the threshold of 0.02, corresponding to the OR gate (Boolean algebra A+B). For the color output and |Δ*A*_416 nm_|, the solution only changes to purple, and the absolute difference of absorbance significantly exceeds the other 3 combinations (logic output 1) when Hg^2+^ is present alone (logic input 1), corresponding to INHIBIT gate (Boolean algebra AB′). When considering the outputs of *A*_264 nm_, *A*_416 nm_, and *A*_530 nm_, the presence of Hg^2+^ alone results in *A*_264 nm_, *A*_416 nm_, and *A*_530 nm_ being below the corresponding threshold (output 0), otherwise output 1, corresponding to the IMPLY gate function (Boolean algebra A′+B).

When ClO^−^ and H_2_O_2_ are introduced as 2 inputs to Au–Ag–Cr NCs, 5 types of logic gates (OR, NAND, INHIBIT, IMPLY, and XOR) can be constructed on the basis of Au–Ag–Cr NCs (Fig. 10C and Fig. S15B). H_2_O_2_ alone causes the solution to turn purple, with a decrease in the absorption peak at 416 nm, which can be attributed to the strong oxidizing property of H_2_O_2_. Similarly, ClO^−^ alone also changes the solution color to purple, increasing the absorption values at 264 and 332 nm, while the absorption peak at 416 nm disappears (Fig. S15B). When ClO^−^ and H_2_O_2_ coexist, the absorption values at 264 and 332 nm decline, and the absorption at 416 nm decreases but does not disappear, likely because of a reaction that occurs between ClO^−^ and H_2_O_2_ to generate Cl^−^, H_2_O, and O_2_. From the perspective of logical calculation to analyze the abovementioned input and output relationship, for the color output, the solution turns purple (logic output 1) if either ClO^−^ or H_2_O_2_ is present (logic input 1), corresponding to the OR gate function (Boolean algebra A+B). The outputs *A*_264 nm_ and *A*_530 nm_ fall below the thresholds of 1.10 or 0.84, respectively, only when both ClO^−^ and H_2_O_2_ are present, which corresponding to the NAND gate function (Boolean algebra A′+B′). Regarding the |Δ*A*_264 nm_| output, the solution's |Δ*A*_264 nm_| is greater than the threshold of 0.03 (logic output 1) only when H_2_O_2_ is present alone, corresponding to the INHIBIT gate function (Boolean algebra A′B); whereas for *A*_332 nm_ and |Δ*A*_332 nm_| outputs, the values exceed the thresholds of 0.82 or 0.04 (logic output 1) only when ClO^−^ is present alone, corresponding to the INHIBIT gate function (Boolean algebra AB′). For the *A*_416 nm_ output, if only ClO^−^ is present, the output is less than the threshold of 0.92 (logic output 0), corresponding to the IMPLY gate function (Boolean algebra A′+B). Furthermore, on the basis of the outputs of |Δ*A*_416 nm_| and |Δ*A*_530 nm_|, XOR gate can be constructed (Boolean algebra A′B+AB′). Interestingly, 2 advanced combinational logic circuits, half-subtractor, can be constructed using ClO^−^ and H_2_O_2_ as 2 inputs, with |Δ*A*_264 nm_| and |Δ*A*_416 nm_| (or |Δ*A*_530 nm_|) serving as 2 outputs (Fig. 10C, light-green markings of the truth table) corresponding to borrow and difference, respectively [see Fig. 10A(c), the green logical notation]. This algorithm process of half-subtractor offers a novel approach for typical molecule-based arithmetic operations and advances the development of sophisticated molecular logic devices.

In addition, using Hg^2+^ and ClO^−^ as inputs, 4 different types of logic gates (OR, YES, AND, and NOT) can similarly be created (Fig. 10D and Fig. S15C). For color output and |Δ*A*_416 nm_|, if either Hg^2+^ or ClO^−^ is present (logic input 1), the solution turns purple, or |Δ*A*_416 nm_| exceeds the threshold of 0.10 (logic output 1), corresponding to the OR gate function (Boolean algebra A+B). For outputs *A*_264 nm_, *A*_332 nm_, and |Δ*A*_332 nm_|, if ClO^−^ is present (logic input 1), the outputs are greater than the thresholds of 1.16, 0.85, or 0.06 (logic output 1), corresponding to the YES gate function (Boolean algebra B); while for |Δ*A*_530 nm_| output, if Hg^2+^ is present (logic input 1), the output is greater than the threshold of 0.06 (logic output 1), also corresponding to the YES gate function (Boolean algebra A). The |Δ*A*_263 nm_| output exceeds the threshold of 0.10 (logic output 1) only when both Hg^2+^ and ClO^−^ are present, corresponding to the AND gate function (Boolean algebra AB). For *A*_416 nm_ and *A*_530 nm_ outputs, the outputs are above the thresholds of 0.80 or 0.90 (logic output 1) only when Hg^2+^ is absent (logic input 0), corresponding to the NOT gate function (Boolean algebra A′). In addition, important transfer gates can be built by combining multiple outputs [*A*_264 nm_ (or *A*_332 nm_ or |Δ*A*_332 nm_|) and |Δ*A*_530 nm_|; Fig. 10D, light blue markings of the truth table). These gates transfer input states to outputs without logical change [0 remains 0, 1 remains 1; input A = output |Δ*A*_530 nm_|, input B = output A_264 nm_ (or *A*_332 nm_ or |Δ*A*_332 nm_|)], making them essential in systems of concatenated logic gates for converting one gate's output into another gate’s input. This concatenation is crucial for performing complex computational operations using molecular logic [29]. In the Au–Ag–Cr NCs system, these 2 gates form a logically reversible system, where each input combination results in a distinct output (Fig. 10D, light blue markings of the truth table). Consequently, using individual outputs (*A*_264 nm_, *A*_332 nm_, |Δ*A*_332 nm_|, or |Δ*A*_530 nm_|) alone would result in information loss. Reversible logic addresses this by ensuring that each input vector corresponds to a distinct output vector, which is increasingly relevant in electronic computers and molecular logic [29]. Therefore, the multichannel and multitarget sensing capabilities of Au–Ag–Cr NCs can facilitate various batch and parallel multifunctional molecular logic computations.

#### Molecular information encoding, cryptography, and steganography based on logic relationships and selective responses of Au–Ag–Cr NCs

**Fig. 11.**
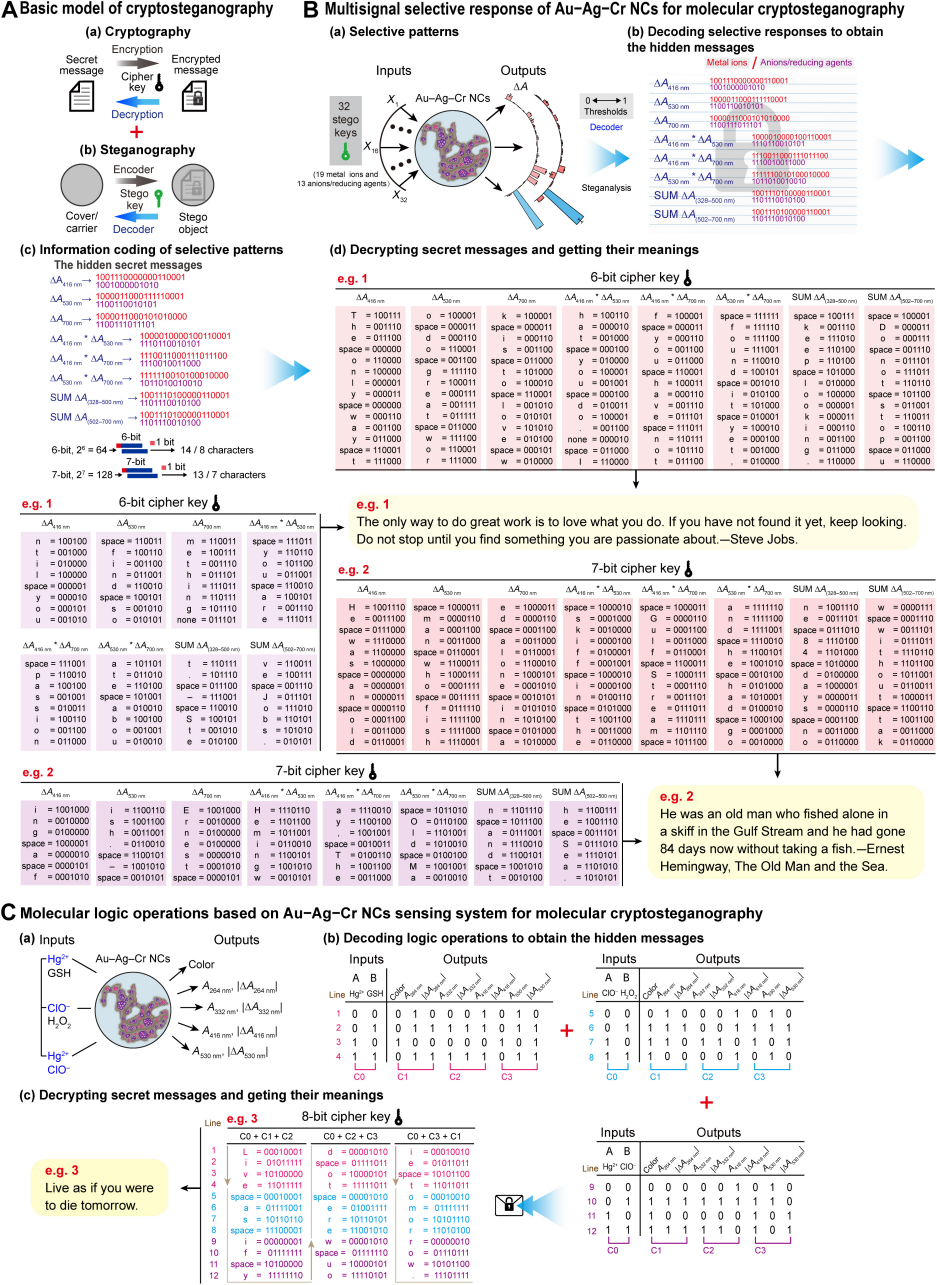
Molecular information encoding, cryptography, and steganography based on logic relationships and selective responses of Au–Ag–Cr NCs. (A) A basic hybrid model of cryptography and steganography, encompassing reversible encryption–decryption and steganalysis processes. (B) Multisignal selective responses of Au–Ag–Cr NCs for molecular cryptosteganography. (a) Au–Ag–Cr NCs (as the stego object) senses 19 metal ions or 13 anions/reducing agents (as stego keys) to generate multisignal outputs (Δ*A*, Δ*A* × Δ*A*, and SUM Δ*A*). (b) The steganalysis process decodes the selective responses to obtain hidden information, represented as 16 lines of the 19-bit and 13-bit binary strings, based on arbitrary thresholds values. (c) Schematic diagram of basic rules for segmenting binary strings and encoding information. (d) Two examples (e.g. 1 and 2) illustrate the decryption process to retrieve secret messages using the correct cipher keys. Yellow text boxes explain the meanings of the secret messages. (C) Input–output logic relationships based on Au–Ag–Cr NCs for molecular cryptosteganography. (a) Au–Ag–Cr NCs (as the stego object) received inputs stimuli (metal ions or active reagents as stego keys) and produce multiresponse outputs (color, *A*_264 nm_, |Δ*A*_264 nm_|, *A*_332 nm_, |Δ*A*_332 nm_|, *A*_416 nm_, |Δ*A*_416 nm_|, *A*_530 nm_, and |Δ*A*_530 nm_|). (b) The steganalysis process decodes the logic operations based on Au–Ag–Cr NCs to obtain hidden information represented as 12 lines of 8-bit binary outputs (derived from Fig. 10B to D). (c) The decryption process retrieves the corresponding secret messages (“Live as if you were to die tomorrow.”) using the cipher keys.

Figure 11A illustrates conventional information protection strategies: cryptography and steganography. Cryptography encodes secret messages into unreadable text for later decryption [Fig. 11A(a)], whereas steganography conceals information within an innocuous carrier [Fig. 11A(b)]. Molecular/nanoscale systems provide novel mechanisms and materials for enhanced security. Integrating both approaches at these scales enables superior information protection [23].

In this study, Au–Ag–Cr NCs nanosystem exhibits selective responsiveness, enabling the encoding, encryption, and concealment of information through steganographic cryptography (Fig. 11B). By adding 19 metal ions and 13 anions or reductants as steganographic keys, Au–Ag–Cr NCs can serve as a steganographic object, producing multisignal selective responses (e.g., color and absorption changes) as outputs [Fig. 11B(a)]. Utilizing distinct threshold values (e.g., “0.018” or “0.012” for Δ*A*_416 nm_, “0” for Δ*A*_530 nm_ and Δ*A*_700 nm_, etc.; Figs. S16 and S17), the selective responses of Au–Ag–Cr NCs across 8 channels are translated into sixteen 19-bit and 13-bit binary strings. This process corresponds to the decoding process, wherein hidden information is retrieved through steganographic analysis [Fig. 11B(a)→(b)]. To decrypt the hidden information encoded in these binary strings, the correct password key is required. Therefore, the 16 rows of 19-bit and 13-bit binary strings are truncated into short binary strings (starting from the first position and shifting one position at a time). This process yields either fourteen 6-bit and eight 6-bit binary strings or thirteen 7-bit and seven 7-bit binary strings, which serve as fundamental encoding units [Fig. 11B(c)]. These 6-bit and 7-bit strings can be used to encode the corresponding number of characters (14 and 8 or 13 and 7). Using simple transposition ciphers or polyalphabetic ciphers, permutations and combinations of 6-bit binary strings (2^6^ = 64; Figs. S18 to S33) or 7-bit binary strings (2^7^ = 128; Figs. S34 to S49) enable encoding of 27, 28, 53, 54, or 55 common characters in a many-to-one manner. Using the correct keys from Figs. S18 to S49, the hidden and encrypted information within the Au–Ag–Cr NCs can be decrypted to reveal the following plaintext [Fig. 11B(d)]: “The only way to do great work is to love what you do. If you have not found it yet, keep looking. Do not stop until you find something you are passionate about.—Steve Jobs.” (From Steve Jobs’ commencement address at Stanford University on 2005 June 12, as shown in, e.g., 1) and “He was an old man who fished alone in a skiff in the Gulf Stream and he had gone 84 days now without taking a fish.—Ernest Hemingway, *The Old Man and the Sea*.” (The opening of the famous novel *The Old Man and the Sea*, setting the stage and introducing the protagonist, as shown in, e.g., 2).

Furthermore, the logic input–output relationships exhibited by Au–Ag–Cr NCs (Fig. 10) can also be employed for encoding, encrypting, and concealing information through molecular cryptographic steganography (Fig. 11C). When adding 3 groups of substances as stego keys, such as Hg^2+^ and GSH, ClO^−^ and H_2_O_2_, or Hg^2+^ and ClO^−^, the Au–Ag–Cr NCs acting as the stego object, produce multisignal responses [such as color, *A*_264 nm_, |Δ*A*_264 nm_|, *A*_332 nm_, |Δ*A*_332 nm_|, *A*_416 nm_, |Δ*A*_416 nm_|, *A*_530 nm_, and |Δ*A*_530 nm_|; Fig. 11C(a)]. By arranging and combining 3 sets of the truth tables [2 inputs + 9 channel outputs; Fig. 11C(b)], a total of 12 rows of 11-bit binary strings can be generated. The above process is equivalent to steganographic analysis [i.e., obtaining hidden information by converting inputs and outputs into binary; Fig. 11C(a) and (b)]. In addition, Fig. 11C(c) shows the specific process of decrypting these 0/1 binary strings into intelligible plaintext. To encode more information, we segmented and combined the 12 rows of 11-bit binary strings obtained from Fig. 11C(b). For example, by combining the 12 rows of 0/1 strings for 2 inputs (represented as C0) with the 12 rows of 0/1 strings for color, *A*_264 nm_, |Δ*A*_264 nm_| 3 outputs (represented as C1), and the 12 rows of 0/1 strings for *A*_332 nm_, |Δ*A*_332 nm_|, *A*_416 nm_ 3 outputs (represented as C2), we obtained 12 rows of 8-bit binary strings (represented as C0 + C1 + C2). Similarly, 2 other combinations were obtained: 8-bit C0 + C2 + C3 and 8-bit C0 + C3 + C1. The 12 rows of 8-bit binary strings can be employed to encode specific characters [Fig. 11C(c)]. Utilizing a simple polyalphabetic cipher, 65 common characters can be encoded in a many-to-one manner through the permutations and combinations of 8 bits (2^8^ = 256) (Figs. S50 to S52). At the same time, on the basis of the 8-bit cipher key, the information encoded in the 12 rows of 8-bit binary strings can be decrypted separately to obtain the following fragmented plaintext: “Live”, “to d”, “ie t” (lines 1 to 4) “ as ”, “ere ”, “omor” (lines 5 to 8) “if y”, “ou w”, and “row” (lines 9 to 12). By merging the individual messages and interpreting their meanings, the corresponding plaintext message can be obtained: "Live as if you were to die tomorrow." [Attributed to the Indian national liberation movement leader Mahatma Gandhi; Fig. 11C(c)]. Thus, the results highlight that Au–Ag–Cr NCs serve as a unique nanocarrier/cover, leveraging their inherent multisignal selective response and input–output logical relationships to provide a molecular framework for information encoding, encryption, and concealment.

## Conclusion

Overall, Au–Ag–Cr NCs synthesized through a simple and efficient method is not only capable of multimodal and multianalyte colorimetric sensing but also plays a role in the field of advanced MIT (including molecular advanced arithmetic logic and reversible logic and information encryption and hiding). Building on the prereaction to generate Au–Cr nanoseeds and by mixing the nanoseeds with Ag^+^, the stabilizer SDBS, and the reducing agent AA, Au–Ag–Cr NCs coated with SDBS were successfully prepared. The morphology of Au–Ag–Cr NCs is characterized by Au/Ag NPs (with an average diameter of 14.15 ± 0.63 nm) attached to the branched Cr NBs (with branch average diameters of 56.93 ± 2.77 nm). By analyzing multisignal changes in color, Tyndall effect, and absorption, Au–Ag–Cr NCs demonstrate the ability to selectively and quantitatively detect 2 kinds of analytes (Hg^2+^ and ClO^−^), and using the combination of signal channels significantly improve selectivity and sensitivity. The addition of Hg^2+^ and ClO^−^ triggers the etching of Au/Ag NPs in the NCs, resulting in changes in color and absorption. Furthermore, by adding different substances as inputs and signal changes of the generating solution as outputs, basic logic gates and advanced logic circuits (half-subtractors and transfer gates) can be constructed. At the same time, through the binary conversion of the multisignal responses, molecular information encoding, encryption, and steganography can be achieved on the basis of the logical relationships and selective responses of Au–Ag–Cr NCs. Compared to previous studies, this research demonstrates the following advantages in the preparation and application of Au–Ag–Cr NCs: (a) updating the preparation mode and comprehensive application scenarios of multicomponent materials to achieve optimized and customized material performance, (b) developing new methods for combining signal channels to improve sensing selectivity and sensitivity, (c) using multichannel responsiveness to expand scale, parallelism, and paradigm of molecular logic computation, and (d) enriching the dimensions and universality of molecular information to enhance the information density and anti-interference capabilities of information safety. In the future, the electrochemical, catalytic, and biological applications of Au–Ag–Cr NCs deserve further attention and exploration. Inspired by this work, the preparation and application of multimetal/multielement nanomaterials will promote the development of material science toward higher levels of integration and functionality. In addition, the fusion of molecular sensing and information technology will burst into greater vitality and promote the development boundary of the new generation of information technology and molecular science.

## Materials and Methods

### Materials and reagents

Potassium dichromate (K_2_Cr_2_O_7_), potassium tetrachloroaurate (KAuCl_4_), sodium borohydride (NaBH_4_), silver nitrate (AgNO_3_), AA, and SDBS were purchased from Aladdin Biochemical Technology Co. Ltd. (Shanghai, China). All aqueous solutions were prepared using ultrapure water produced by a Milli-Q system (Millipore, USA) with a resistivity of 18.2 MΩ·cm.

### Preparation and purification of Au–Ag–Cr NCs

The synthesis procedure, optimized under the established reaction conditions, is described as follows. First, Au–Cr nanoseeds were prepared by sequentially mixing Cr^6+^, Au^3+^, and NaBH_4_ in an ice bath for 15 min, followed by a 15-min rest at room temperature and centrifugation for resuspension [28]. The preparation steps of the Au–Ag or Ag–Cr nanoseeds were consistent. Then, 250 μl of Au–Cr nanoseeds (1:2 mM), 175 μl of AgNO_3_ (10 mM), and 175 μl of AA (10 mM) were sequentially added to 400 μl of SDBS (10 mM), adjusting the total volume to 1,000 μl. Each reactant was mixed thoroughly by pipetting 3 to 5 times. After a 70-min reaction at room temperature, the mixture was centrifuged and suspended twice. The final concentration of Au–Ag–Cr NCs was determined by the final concentration of the metal precursors added to the reaction mixture, specifically 0.25 mM Au, 1.75 mM Ag, and 0.5 mM Cr.

### Characterization of Au–Ag–Cr NCs and their mixtures with Hg^2+^ or ClO^−^

The optical properties of the purified Au–Ag–Cr NCs were assessed using a SpectraMax M5 microplate reader (Molecular Devices, USA) to measure their absorption spectra. The morphological and compositional features of the Au–Ag–Cr NCs, as well as their mixtures with Hg^2+^/ClO^−^, were examined using a FEI Talos F200X TEM (FEI, USA) equipped with an EDS. The crystalline phase, surface functional groups, and elemental composition of the NCs were further characterized using an XRD (Rigaku, Japan), an IS-50 FTIR (Thermo Fisher Scientific, USA), and a 250Xi XPS (Thermo Fisher Scientific, USA), respectively.

### Selective and quantitative detection of Hg^2+^ or ClO^−^ based on Au–Ag–Cr NCs

Au–Ag–Cr NCs can be used for 2 distinct types of analytes: metal ions and anions/reducing agents. Metal ions or anions/reducing agents (100 μM) were introduced to Au–Ag–Cr NCs (0.25:1.75:0.5 mM, diluted 1.33 times). Following a 5-min reaction at room temperature, the color, Tyndall effect, and UV-visible absorption spectra of the resultant solutions were recorded. Single-channel absorbance changes at specific wavelengths were quantified by calculating the absorbance differences at 416, 530, and 700 nm (Δ*A*_416 nm_, Δ*A*_530 nm_, and Δ*A*_700 nm_) between the mixture and the Au–Ag–Cr NCs alone. These changes across different channels were subsequently integrated [Δ*A*_416 nm_ × Δ*A*_530 nm_, Δ*A*_416 nm_ × Δ*A*_700 nm_, Δ*A*_530 nm_ × Δ*A*_700 nm_, SUM Δ*A*_(328–500 nm)_, and SUM Δ*A*_(502–700 nm)_] to assess improvements in selectivity and sensitivity. Selectivity for a specific channel was determined by calculating the ratio of the response to Hg^2+^ and ClO^−^ compared to the second highest response of an interfering substance.

For quantitative detection of Hg^2+^ or ClO^−^, we mixed Au–Ag–Cr NCs with concentrations of 0 to 450 μM Hg^2+^ or ClO^−^, respectively. After a 5-min reaction at room temperature, the color, Tyndall effect, and UV-visible absorption spectra of the solution were recorded. We analyzed the correlation between signal changes and concentrations of Hg^2+^ or ClO^−^ in both single and combined channels. The LOD was determined by calculating 3 times the standard deviation of the blank signal divided by the slope of the calibration curve.

### Actual sample analysis

Pond water and tap water samples were collected from campus for spiking and recovery experiments with Hg^2+^ or ClO^−^. The samples were filtered through a 0.22-μm membrane and used to dilute standard Hg^2+^ or ClO^−^ solutions. Known concentrations of Hg^2+^ or ClO^−^ were then spiked into the Au–Ag–Cr NC solution (0.25:1.75:0.5 mM, diluted 1.33 times). After a 5-min reaction at room temperature, the mixed solutions were analyzed across different signal channels.

### Molecular logic operations based on the response of Au–Ag–Cr NCs to Hg^2+^ and ClO^−^

By setting different substances added to Au–Ag–Cr NCs (such as Hg^2+^, ClO^−^, GSH, or H_2_O_2_) as inputs and employing colorimetric changes across multiple signal channels (color, absorbance, and their absolute differences at 264, 332, 416, and 530 nm) as outputs, a range of basic logic gates and advanced cascaded logic circuits can be constructed. The substances were mixed in different combinations and reacted at room temperature for 10 min. The color and absorption spectra of the resulting solutions were then photographed or measured. Ultimately, by establishing appropriate thresholds, diverse Boolean logic gates and complex molecular logic circuits can be constructed.

### Molecular cryptosteganography based on Au–Ag–Cr NCs

In molecular cryptosteganography leveraging the selective responses of Au–Ag–Cr NCs, 19 metal ions or 13 anions/reducing agents (acting as stego keys) were introduced to the Au–Ag–Cr NCs (the stego object), eliciting selective responses across single and combined channels. The response signals from 8 channels were then converted into 8 sets of 19-bit or 13-bit binary strings based on predefined thresholds. These binary strings were then split into fourteen 6-bit or thirteen 7-bit strings from the 19-bit strings and eight 6-bit or seven 7-bit strings from the 13-bit strings. Last, these binary strings were decrypted into understandable information using password tables.

All experimental operations followed the same procedure as in the selectivity of Au–Ag–Cr NCs.

In molecular cryptosteganography based on molecular logic operations of Au–Ag–Cr NCs, different combinations of Hg^2+^ and GSH, or ClO^−^ and H_2_O_2_, or Hg^2+^ and ClO^−^ were used as stego keys. These combinations were added to the Au–Ag–Cr NCs (the stego objects), which produced multisignal outputs, including color changes, absorbance shifts, and absolute differences at 264, 332, 416, and 530 nm. By analyzing these outputs, these groups of truth tables (each with 2 inputs and 9 outputs) were generated from the 4-input combinations. These outputs were designated as C0, C1, C2, and C3, respectively. By combining these outputs (C0 + C1 + C2, C0 + C2 + C3, and C0 + C3 + C1), 3 sets of 12 lines of 8-bit binary strings were generated for encoding more information. These binary strings were then decrypted into understandable information using corresponding password tables. The experimental procedures followed the same protocol as those used in the molecular logic gate studies based on Au–Ag–Cr NCs.

## Data Availability

All data required to support the conclusions are presented in the main text and/or the Supplementary Materials.
