# Supplementary material for: Multifunctional Au–Ag–Cr Nanocomposites: From Multiplex Sensing and Advanced Logic Computing to Scalable Information Protection
Source: Research (Wash D C). 2025 Jul 22;8:0763. doi: 10.34133/research.0763 (PMC12280986; doi:10.34133/research.0763)
Supplement: Supplementary 1 — Figs. S1 to S52 Tables S1 and S2 [file research.0763.f1.docx]

**Supporting Information**

**Multifunctional Trimetallic Gold-Silver-Chromium Nanocomposites from Multimode and Multi-Analyte Sensing, Advanced Arithmetic and Reversible Logic to Long-Text Information Protection**

Jie Zhou^a, b, #^, Jiao Yang Lu^a, b, #,^ *, Zhi Xin Xie^b^, and Wei Tao Huang ^b,^ *

*^a^ Hunan key laboratory of the research and development of novel pharmaceutical preparations, Hunan Provincial University Key Laboratory of the Fundamental and Clinical Research on Functional Nucleic Acid, “The 14th Five-Year Plan” Application Characteristic Discipline of Hunan Province (Clinical Medicine), School of Nursing, Changsha Medical University, Changsha 410219, P. R. China*

*^b^* *State Key Laboratory of Developmental Biology of Freshwater Fish, Hunan Provincial Key Laboratory of Microbial Molecular Biology, College of Life Science, Hunan Normal University, Changsha 410081, P. R. China*

^#^ These authors contributed equally to this work.

*Corresponding author:

E-mail: joyerlu@qq.com. Fax: (+86)731-8860-2602; Tel: (+86)731-8860-2602

E-mail: vthuang@hunnu.edu.cn. Fax: (+86)731-8887-2905; Tel: (+86)731-8887-2905

1. **Preparation and optimization of Au−Ag−Cr** **NCs**


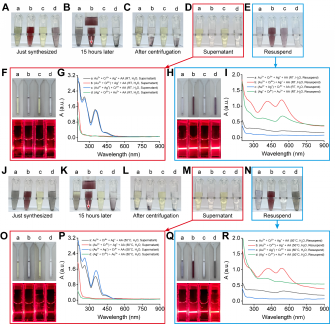


**Figure S1.** Exploration of the synthesis of Au−Ag−Cr NCs using two methods (one-pot reduction and nanoseed) under different conditions (A-I for room temperature (RT) and J-R for 50 °C heating) and AA as the reductant. (A-E, J-N) Color photos of different solution combinations at different operational nodes, namely, just after synthesis (A, J), after standing for 15 hours (B, K, enlarged insets and red arrows showed liquid layering and precipitation of b), after centrifugation (C, L), the supernatant after centrifugation (D, M), and resuspension of the precipitate after centrifugation (E, N). (F-I, O-R) The color and Tyndall effect photos, and UV-visible absorbance spectra of the supernatant (F, G, O, P) and precipitate resuspension (H, I, Q, R) obtained from different solution combinations.


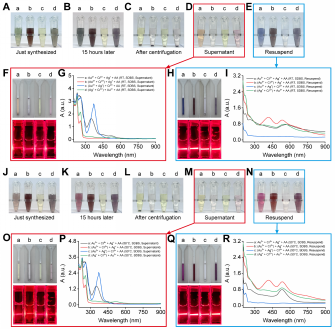


**Figure S2.** Exploration of the synthesis of Au−Ag−Cr NCs using two methods (one-pot reduction and nanoseed) under different conditions (A-I for room temperature (RT) and J-R for 50 °C heating), and AA as the reductant, and SDBS as the stabilizer. (A-E, J-N) Color photos of different solution combinations at different operational nodes, namely, just after synthesis (A, J), after standing for 15 hours (B, K), after centrifugation (C, L), the supernatant of centrifugation (D, M), and resuspension of the precipitate after centrifugation (E, N). (F-I, O-R) The color and Tyndall effect photos, and UV-visible absorbance spectra of the supernatant (F, G, O, P) and precipitate resuspension (H, I, Q, R) obtained from different solution combinations.


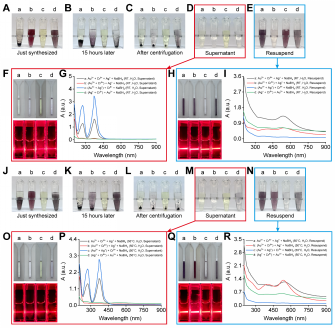


**Figure S3.** Exploration of the synthesis of Au−Ag−Cr NCs using two methods (one-pot reduction and nanoseed) under different conditions (A-I room temperature (RT) and J-R 50 °C heating) and NaBH_4_ as the reductant. (A-E, J-N) Color photos of different solution combinations at different operational nodes, namely, just after synthesis (A, J), after standing for 15 hours (B, K), after centrifugation (C, L), the supernatant after centrifugation (D, M), and resuspension of the precipitate after centrifugation (E, N). (F-I, O-R) The color and Tyndall effect photos, and UV-visible absorbance spectra of the supernatant (F, G, O, P) and precipitate resuspension (H, I, Q, R) obtained from different solution combinations.


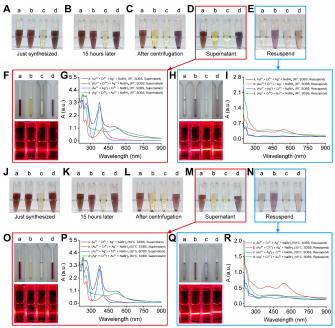


**Figure S4.** Exploration of the synthesis of Au−Ag−Cr NCs using two methods (one-pot reduction and nanoseed) under different conditions (A-I for room temperature (RT) and J-R for 50 °C heating), NaBH_4_ as the reductant, and SDBS as the stabilizer. (A-E, J-N) Color photos of different solution combinations at different operational nodes, namely, just after synthesis (A, J), after standing for 15 hours (B, K), after centrifugation (C, L), the supernatant after centrifugation (D, M), and resuspension of the precipitate after centrifugation (E, N). (F-I, O-R) The color and Tyndall effect photos, and UV-visible absorbance spectra of the supernatant (F, G, O, P) and precipitate resuspension (H, I, Q, R) obtained from different solution combinations.

1. **Exploration of the optimal reaction time for preparing Au−Ag−Cr** **NCs.**


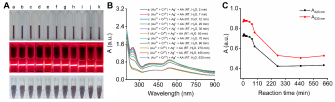


**Figure S5.** Exploring the time-dependent kinetics of the mixed reaction solution using the Au−Cr nanoseed method + Ag^+^ + AA, at room temperature, H_2_O as the solvent. (A, B) Color and Tyndall effect photos (A) and UV-visible absorption spectra (B) of the mixed solution after reacting for different times. (C) Changes in the absorbance at 420 nm (characteristic absorption peak of Ag NPs) and 530 nm (characteristic absorption peak of Au NPs) of the mixed solution after reacting for different times.


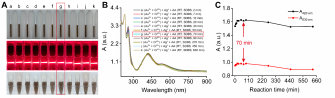


**Figure S6.** Exploring the time-dependent kinetics of the mixed reaction solution using the Au−Cr nanoseed method + Ag^+^ + AA, at room temperature, SDBS as the stabilizer. (A, B) Color and Tyndall effect photos (A) and UV-visible absorption spectra (B) of the mixed solution after reacting for different times. (C) Changes in the absorbance at 420 nm (characteristic absorption peak of Ag NPs) and 530 nm (characteristic absorption peak of Au NPs) of the mixed solution after reacting for different times.


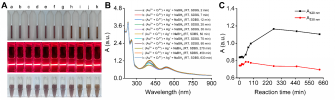


**Figure S7.** Exploring the time-dependent kinetics of the mixed reaction solution using the Au−Cr nanoseed method + Ag^+^ + NaBH_4_, at room temperature, SDBS as the stabilizer. (A, B) Color and Tyndall effect photos (A) and UV-visible absorption spectra (B) of the mixed solution after reacting for different times. (C) Changes in the absorbance at 420 nm (characteristic absorption peak of Ag NPs) and 530 nm (characteristic absorption peak of Au NPs) of the mixed solution after reacting for different times.

1. **Exploration of the concentration ratio of each addition in Au−Ag−Cr** **NCs.**


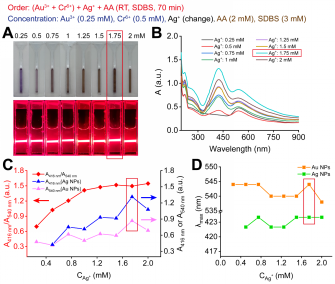


**Figure S8.** Exploring the effect of the altered concentration of Ag^+^ on the synthesis of Au−Ag−Cr ternary nanomaterials. Note: The synthesis order was (Au^3+^ + Cr^6+^) + Ag^+^ + AA (RT, SDBS, 70 min). In the expression of synthetic concentration, blue font indicated the determined concentration, black represented concentration changes and brown font indicated undetermined. (A, B) Color and Tyndall effect photos (A) and UV-visible absorption spectra (B) of Au−Ag−Cr NCs synthesized at different AgNO_3_ addition concentrations. (C, D) Changes in the characteristic absorbance and its ratio (C), and the maximum absorption wavelength (D) of Au/Ag NPs in Au−Ag−Cr NCs synthesized at different AgNO_3_ addition concentrations.


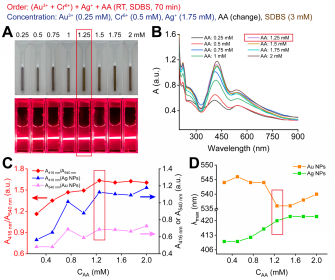


**Figure S9.** Exploring the effect of the altered concentration of the AA on the synthesis of Au−Ag−Cr ternary nanomaterials. Note: The synthesis order is (Au^3+^ + Cr^6+^) + Ag^+^ + AA (RT, SDBS, 70 min). In the expression of synthetic concentration, blue font indicated the determined concentration, black represented concentration changes and brown font indicated undetermined. (A, B) Color and Tyndall effect photos (A) and UV-visible absorption spectrum (B) of Au−Ag−Cr NCs synthesized at different AA addition concentrations. (C, D) Changes in the characteristic absorbance and its ratio (C), and the maximum absorption wavelength (D) of Au/Ag NPs in Au−Ag−Cr NCs synthesized at different AA addition concentrations.


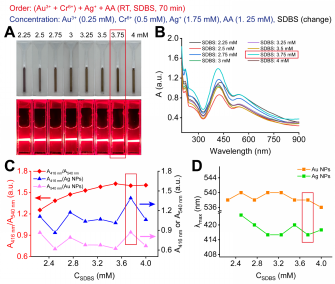


**Figure S10.** Exploring the effect of the concentration of SDBS on the synthesis of Au−Ag−Cr ternary nanomaterials. Note: The synthesis order is (Au^3+^ + Cr^6+^) + Ag^+^ + AA (RT, SDBS, 70 min). In the expression of synthetic concentration, blue font indicated the determined concentration, black represented concentration changes. (A, B) Color and Tyndall effect photos (A) and UV-visible absorption spectrum (B) of Au−Ag−Cr NCs synthesized at different SDBS addition concentrations. (C, D) Changes in the characteristic absorbance and its ratio (C), and the maximum absorption wavelength (D) of Au/Ag NPs in Au−Ag−Cr NCs synthesized at different SDBS addition concentrations.

1. **The storage stability of Au−Ag−Cr NCs.**

**
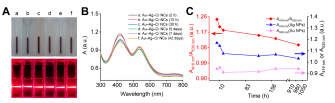
**

**Figure S11.** **The storage stability of Au−Ag−Cr NCs.** (A-C) The color and Tyndall effect photos (A), absorption spectra (B), and changes in the characteristic absorbance and its ratio (C) of Au/Ag NPs in Au−Ag−Cr NCs under different storage times. Au−Ag−Cr NCs, with a molar ratio of 0.25: 1.75: 0.5 mM, without dilution.

1. **Kinetics of the reaction of Au−Ag−Cr NCs with Hg^2+^ and ClO^−^.**


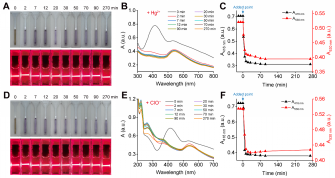


**Figure S12. Kinetics of the reaction of Au−Ag−Cr NCs with Hg^2+^ and ClO^−^.** (A, D) Color and Tyndall effect photos, (B, E) UV-visible absorption spectra, and (C, F) changes in the characteristic absorbance of Au/Ag NPs of Au−Ag−Cr NCs after reaction with Hg^2+^ or ClO^−^ at different times. Au−Ag−Cr NCs, 0.25: 1.75: 0.5 mM, diluted 1.33 fold; Hg^2+^ and ClO^−^: 100 μM.

1. **Anti-interference ability of Au−Ag−Cr NCs.**


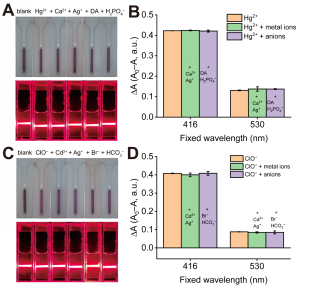


**Figure S13.** The anti-interference ability of Au−Ag−Cr NCs. Comparison of solution color, Tyndall effect (A, C), and absorption responses at 416 nm and 530 nm (B, D) of Au−Ag−Cr NCs to Hg^2+^ (A, B) or ClO^−^ (C, D) and its coexistence with other metal ions or anions. Au−Ag−Cr NCs, 0.25: 1.75: 0.5 mM, diluted 1.33 fold; Metal ions and anions/reductants: 60 μM.

1. **Comparison of the detection limits and linear ranges of other reported Hg^2+^** **assays.**

**Table S1**. Comparison of the detection limits and linear ranges of other reported Hg^2+^ assays.

| Probe | Method | Detection limit  (nM) | Linear range  (μM) | Ref |
| --- | --- | --- | --- | --- |
| Ag@Au NPs | Colorimetry | 5 | 0.03~4 | 1 |
| C-Au@Ag-NPs | Colorimetry | 0.7 | 0.09~90 | 2 |
| Au-Ag core-shell nanorods | Colorimetry | 273 | 1~60 | 3 |
| Ag NPRs ^a^ | Colorimetry | 3 | 0.003~0.5 | 4 |
| MoS_2_-Au composites | Colorimetry | 5 | 0.02~20 | 5 |
| AgTrngs ^b^ | Colorimetry | 4 | 0.01~50 | 6 |
| Tailor-made fluorogenic probes | Fluorimetry | 40 | 0~10 | 7 |
| Pyrazoline-based fluorescence sensor | Fluorimetry | 14.54 μM | 20-200 | 8 |
| NSCDs ^c^ | Fluorimetry | 6.5 | 0.01~0.25 | 9 |
| Zr-DMBD MOFs/3D-KSC ^d^ | Electrochemical | 50 | 0.25~3.5 | 10 |
| Au−Ag−Cr NCs | Colorimetry | 39 (ΔA_416 nm_)  203 (ΔA_530 nm_)  167 (ΔA_700 nm_)  0.027 (ΔA_416 nm_ *ΔA_530 nm_)  0.018 (ΔA_416 nm_ *ΔA_700 nm_)  0.183 (ΔA_530 nm_ *ΔA_700 nm_)  646 (SUM ΔA_(328-500 nm)_)  2.16 μM (SUM ΔA_(502-700 nm)_) | 0.125~90, 90~450  0.125~60, 60~450  0.125~90, 150~450  0.25~90, 90~330  0.5~90, 90~450  0.125~60, 90~450  0.125~60, 60~450  0.125~90, 90~450 | This work |

^a^ Ag NPRs: Silver nanoprisms.

^b^ AgTrngs: Silver nanotriangles.

^c^ NSCDs: Fluorescent nitrogen and sulfur co-doped carbon dots.

^d^ Zr-DMBD MOFs/3D-KSC: Zr (IV) and 2,5-dimercaptoterephthalic acid (Zr-DMBD MOFs) and attached to three-dimensional kenaf stem-derived carbon (3D-KSC).

1. **Comparison of the detection limits and linear ranges of other reported optical ClO**^−^ **assays.**

**Table S2**. Comparison of the detection limits and linear ranges of other reported optical ClO^−^ assays.

| Probe | Method | Detection limit  (nM) | Linear range  (μM) | Ref |
| --- | --- | --- | --- | --- |
| RD-CDs ^a^ | Colorimetry | 83 | 0.1~100 | 11 |
| CPB-AgNPs ^b^ | Colorimetry | 8.97μM | 9.72~87.48 | 12 |
| Tricyanoethylene-derived colorimetric probe | Colorimetry | 4.0 μM | 0~140 | 13 |
| MDT ^c^ | Colorimetry | 5.67 μM | 0~70 | 14 |
| Au NS@Ag NRs ^d^ | Colorimetry | 240 | 0.5~30 | 15 |
| BMH-2∙Cl ^e^ | Colorimetry  Fluorimetry | 2.4 μM  290 | 0~50  0~6 | 16 |
| FH-GA-CQDs ^f^ | Fluorimetry | 93 | 0~70 | 17 |
| Dual-site fluorescent probe Geisha-1 | Fluorimetry | 28.2 | 0~150 | 18 |
| CD/CCM@ZIF-8 ^g^ | Fluorimetry | 67 | 0.1~50 | 19 |
| AuNRs@UCNPs ^h^ | Fluorimetry  Colorimetry | 156  231 | 0.5~140  1~180 | 20 |
| Au−Ag−Cr NCs | Colorimetry | 43 (ΔA_416 nm_)  449 (ΔA_530 nm_)  200 (ΔA_700 nm_)  0.043 (ΔA_416 nm_ *ΔA_530 nm_)  0.026 (ΔA_416 nm_ *ΔA_700 nm_)  0.196 (ΔA_530 nm_ *ΔA_700 nm_)  2.17 μM (SUM ΔA_(328-500 nm)_)  3.65 μM (SUM ΔA_(502-700 nm)_) | 0.25~90, 90~450  1~210  0.25~150  15~210  15~210  15~330  0.25~210  0.125~210 | This work |

^a^ RD-CDs: Novel carbon dots.

^b^ CPB-AgNPs: Cetylpyridinium bromide (CPB) capped silver nanoparticles.

^c^ MDT: A novel dual-responsive colorimetric/fluorescent probe.

^d^ Au NS@Ag NRs: Au nanospheres (NSs)@Ag nanorods (NRs).

^e^ BMH-2∙Cl: 2,2 '-(((1E,1 ' E)-[2,2 '-bithiophene]- 5,5 ' diylbis(methanylylidene))bis(hydrazin-1-yl-2-ylidene))bis(N,N,N-trimethyl-2-oxoethan-1-aminium) chloride.

^f^ FH-GA-CQDs: Surface modification of carbon quantum dots by fluorescein.

^g^ CD/CCM@ZIF-8: Carbon dot/curcumin zeolitic imidazolate framework-8.

^h^ AuNRs@UCNPs: Gold nanorods and lanthanide doped up-conversion nanoparticles

1. **Real water sample analysis.**

**
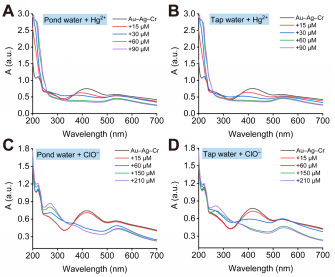
**

**Figure S14.** Absorption spectra of Au−Ag−Cr NCs before and after the addition of Hg^2+^ or ClO^−^ to the pond water (A, C) and tap water (B, D). Au−Ag−Cr NCs, 0.25: 1.75: 0.5 mM, diluted by 1.33-fold; Hg^2+^ and ClO^−^: 15, 60, 90, 150 or 210 μM.

1. **Molecular logic computation based on Au−Ag−Cr NCs multimode colorimetric sensing system.**

**
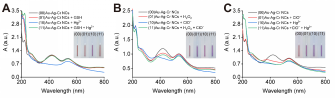
**

**Figure S15.** Absorption spectra of different input combinations: (A) GSH and Hg^2+^ combination, (B) H_2_O_2_ and ClO^−^ combination, (C) Hg^2+^ and ClO^−^ combination. Insets of A-C: color photos of the corresponding combination solutions. Au−Ag−Cr NCs, 0.25: 1.75: 0.5 mM; GSH and H_2_O_2_: 10 mM, Hg^2+^ and ClO^−^: 60 μM. Reaction time: 10 min.

1. **Molecular information encoding, cryptography, and steganography based on logic relationships and selective responses of Au−Ag−Cr NCs.**


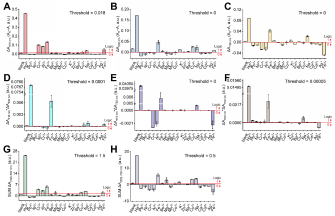


**Figure S16.** The selective response modes of the Au−Ag−Cr NCs nanosystem to 19 metal ions was converted to 0/1 binary strings. The red lines represent the threshold lines. The thresholds are set as follows: ΔA_416 nm_ at 0.018 a.u. (A), ΔA_530 nm_ at 0 a.u. (B), ΔA_700 nm_ at 0 a.u. (C), ΔA_416 nm_ * ΔA_530 nm_ at 0.0001 a.u. (D), ΔA_416 nm_ * ΔA_700 nm_ at 0 a.u. (E), ΔA_530 nm_ * ΔA_700 nm_ at 0.00005 a.u. (F), SUM ΔA_(328 nm-500 nm)_ at 1.5 a.u. (G), SUM ΔA_(502 nm-700 nm)_ at 0.5 a.u. (H).

**
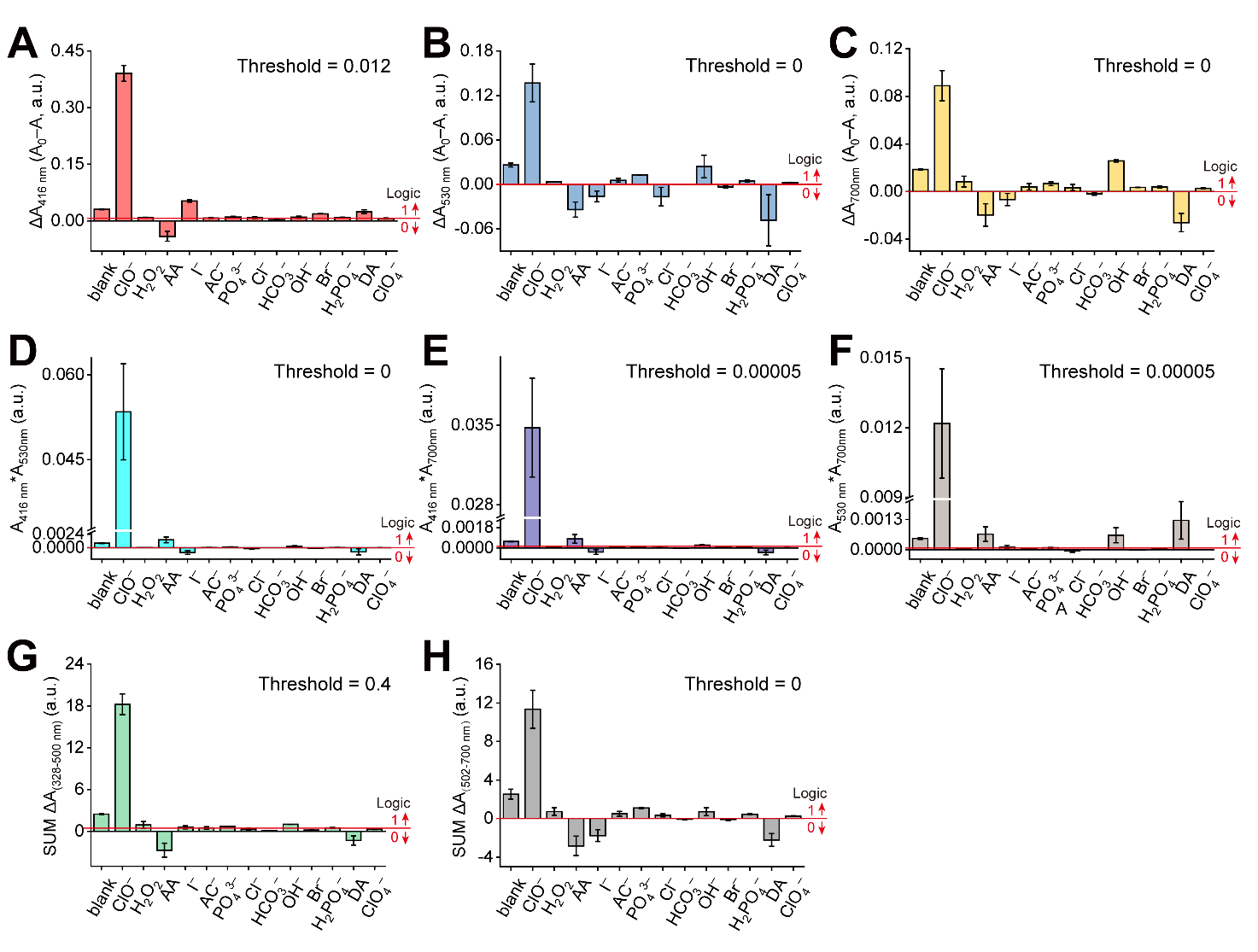
**

**Figure S17.** The selective response modes of the Au−Ag−Cr NCs nanosystem to 13 anions or reducing agents was converted to 0/1 binary string. The red lines represent the threshold lines. The thresholds are set as follows: ΔA_416 nm_ at 0.012 a.u. (A), ΔA_530 nm_ at 0 a.u. (B), ΔA_700 nm_ at 0 a.u. (C), ΔA_416 nm_ * ΔA_530 nm_ at 0 a.u. (D), ΔA_416 nm_ * ΔA_700 nm_ at 0.00005 a.u. (E), ΔA_530 nm_ * ΔA_700 nm_ at 0.00005 a.u. (F), SUM ΔA_(328 nm-500 nm)_ at 0.4 a.u. (G), SUM ΔA_(502 nm-700 nm)_ at 0 a.u. (H).

**
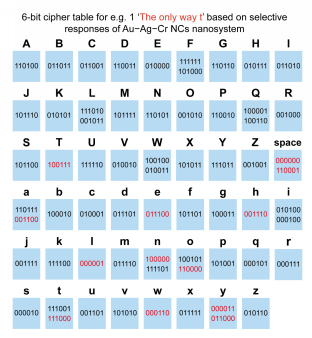
**

**Figure S18.** 6-bit cipher table for e.g. 1 ‘The only way t’ based on selective responses (ΔA_416 nm_) of Au−Ag−Cr NCs nanosystem to metal ions for encoding 53 printable ASCII characters. The colored 6-bit binary strings represent the corresponding keys used to decode hidden information in a response system based on Au−Ag−Cr NCs.

**
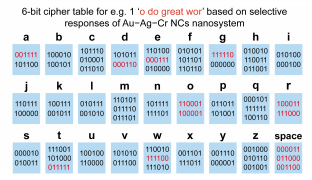
**

**Figure S19.** 6-bit cipher table for e.g. 1 ‘o do great wor’ based on selective responses (ΔA_530 nm_) of Au−Ag−Cr NCs nanosystem to metal ions for encoding 27 printable ASCII characters. The colored 6-bit binary strings represent the corresponding keys used to decode hidden information in a response system based on Au−Ag−Cr NCs.

**
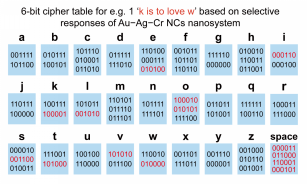
**

**Figure S20.** 6-bit cipher table for e.g. 1 ‘k is to love w’ based on selective responses (ΔA_700 nm_) of Au−Ag−Cr NCs nanosystem to metal ions for encoding 27 printable ASCII characters. The colored 6-bit binary strings represent the corresponding keys used to decode hidden information in a response system based on Au−Ag−Cr NCs.

**
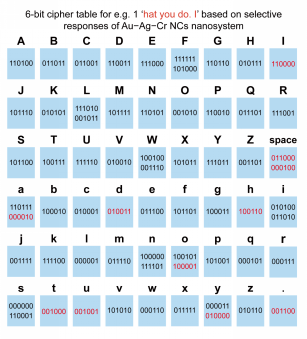
**

**Figure S21.** 6-bit cipher table for e.g. 1 ‘hat you do. I’ based on selective responses (ΔA_416 nm_ *ΔA_530 nm_) of Au−Ag−Cr NCs nanosystem to metal ions for encoding 54 printable ASCII characters. The colored 6-bit binary strings represent the corresponding keys used to decode hidden information in a response system based on Au−Ag−Cr NCs.

**
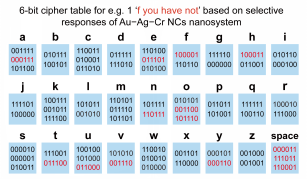
**

**Figure S22.** 6-bit cipher table for e.g. 1 ‘f you have not’ based on selective responses (ΔA_416 nm_ *ΔA_700 nm_) of Au−Ag−Cr NCs nanosystem to metal ions for encoding 27 printable ASCII characters. The colored 6-bit binary strings represent the corresponding keys used to decode hidden information in a response system based on Au−Ag−Cr NCs.

**
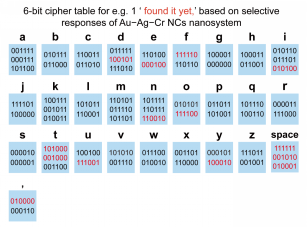
**

**Figure S23.** 6-bit cipher table for e.g. 1 ‘ found it yet,’ based on selective responses (ΔA_530 nm_ *ΔA_700 nm_) of Au−Ag−Cr NCs nanosystem to metal ions for encoding 28 printable ASCII characters. The colored 6-bit binary strings represent the corresponding keys used to decode hidden information in a response system based on Au−Ag−Cr NCs.

**
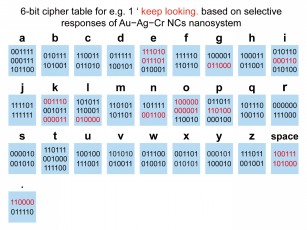
**

**Figure S24.** 6-bit cipher table for e.g. 1 ‘ keep looking.’ based on selective responses (SUM ΔA_(328-500 nm)_) of Au−Ag−Cr NCs nanosystem to metal ions for encoding 28 printable ASCII characters. The colored 6-bit binary strings represent the corresponding keys used to decode hidden information in a response system based on Au−Ag−Cr NCs.

**
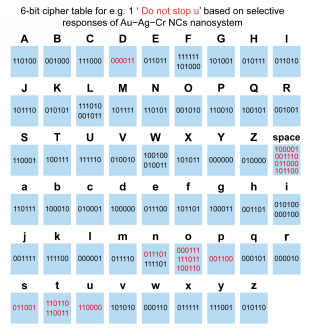
**

**Figure S25.** 6-bit cipher table for e.g. 1 ‘Do not stop u’ based on selective responses (SUM ΔA_(502-700 nm)_) of Au−Ag−Cr NCs nanosystem to metal ions for encoding 53 printable ASCII characters. The colored 6-bit binary strings represent the corresponding keys used to decode hidden information in a response system based on Au−Ag−Cr NCs.

**
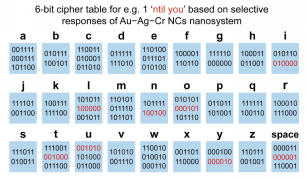
**

**Figure S26.** 6-bit cipher table for e.g. 1 ‘ntil you’ based on selective responses (ΔA_416 nm_) of Au−Ag−Cr NCs nanosystem to anions or reducing agents for encoding 27 printable ASCII characters. The colored 6-bit binary strings represent the corresponding keys used to decode hidden information in a response system based on Au−Ag−Cr NCs.

**
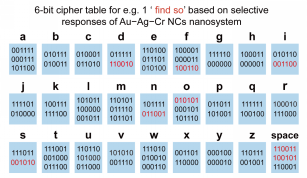
**

**Figure S27.** 6-bit cipher table for e.g. 1 ‘ find so’ based on selective responses (ΔA_530 nm_) of Au−Ag−Cr NCs nanosystem to anions or reducing agents for encoding 27 printable ASCII characters. The colored 6-bit binary strings represent the corresponding keys used to decode hidden information in a response system based on Au−Ag−Cr NCs.

**
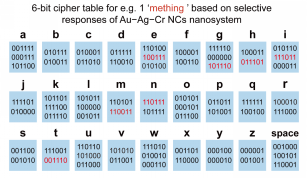
**

**Figure S28.** 6-bit cipher table for e.g. 1 ‘mething ’ based on selective responses (ΔA_700 nm_) of Au−Ag−Cr NCs nanosystem to anions or reducing agents for encoding 27 printable ASCII characters. The colored 6-bit binary strings represent the corresponding keys used to decode hidden information in a response system based on Au−Ag−Cr NCs.

**
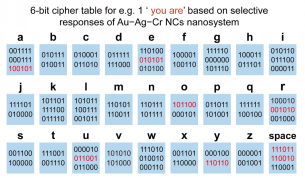
**

**Figure S29.** 6-bit cipher table for e.g. 1 ‘ you are’ based on selective responses (ΔA_416 nm_ *ΔA_530 nm_) of Au−Ag−Cr NCs nanosystem to anions or reducing agents for encoding 27 printable ASCII characters. The colored 6-bit binary strings represent the corresponding keys used to decode hidden information in a response system based on Au−Ag−Cr NCs.

**
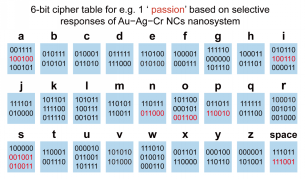
**

**Figure S30.** 6-bit cipher table for e.g. 1 ‘ passion’ based on selective responses (ΔA_416 nm_ *ΔA_700 nm_) of Au−Ag−Cr NCs nanosystem to anions or reducing agents for encoding 27 printable ASCII characters. The colored 6-bit binary strings represent the corresponding keys used to decode hidden information in a response system based on Au−Ag−Cr NCs.

**
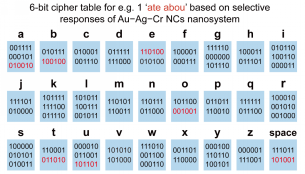
**

**Figure S31.** 6-bit cipher table for e.g. 1 ‘ate abou’ based on selective responses (ΔA_530 nm_ *ΔA_700 nm_) of Au−Ag−Cr NCs nanosystem to anions or reducing agents for encoding 27 printable ASCII characters. The colored 6-bit binary strings represent the corresponding keys used to decode hidden information in a response system based on Au−Ag−Cr NCs.

**
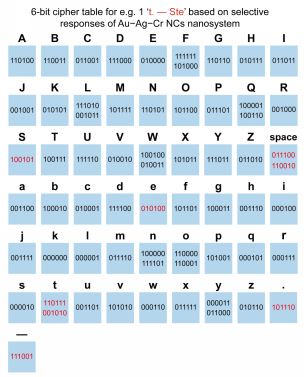
**

**Figure S32.** 6-bit cipher table for e.g. 1 ‘t. − Ste’ based on selective responses (SUM ΔA_(328-500 nm)_) of Au−Ag−Cr NCs nanosystem to anions or reducing agents for encoding 55 printable ASCII characters. The colored 6-bit binary strings represent the corresponding keys used to decode hidden information in a response system based on Au−Ag−Cr NCs.

**
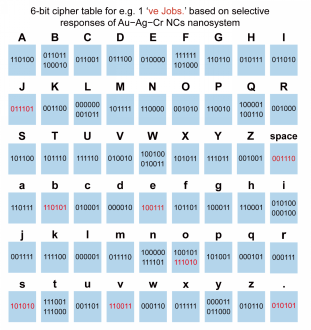
**

**Figure S33.** 6-bit cipher table for e.g. 1 ‘ve Jobs.’ based on selective responses (SUM ΔA_(502-700 nm)_) of Au−Ag−Cr NCs nanosystem to anions or reducing agents for encoding 54 printable ASCII characters. The colored 6-bit binary strings represent the corresponding keys used to decode hidden information in a response system based on Au−Ag−Cr NCs.

**
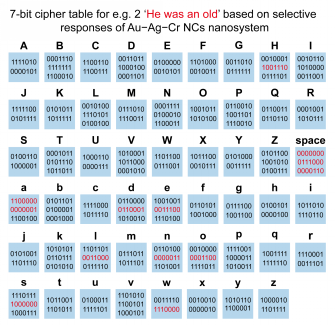
**

**Figure S34.** 7-bit cipher table for e.g. 2 ‘He was an old’ based on selective responses (ΔA_416 nm_) of Au−Ag−Cr NCs nanosystem to metal ions for encoding 53 printable ASCII characters. The colored 7-bit binary strings represent the corresponding keys used to decode hidden information in a response system based on Au−Ag−Cr NCs.

**
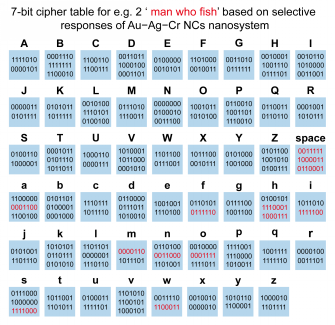
**

**Figure S35.** 7-bit cipher table for e.g. 2 ‘ man who fish’ based on selective responses (ΔA_530 nm_) of Au−Ag−Cr NCs nanosystem to metal ions for encoding 53 printable ASCII characters. The colored 7-bit binary strings represent the corresponding keys used to decode hidden information in a response system based on Au−Ag−Cr NCs.

**
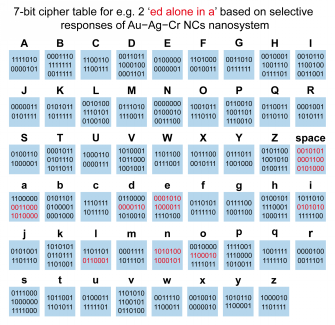
**

**Figure S36.** 7-bit cipher table for e.g. 2 ‘ed alone in a’ based on selective responses (ΔA_700 nm_) of Au−Ag−Cr NCs nanosystem to metal ions for encoding 53 printable ASCII characters. The colored 7-bit binary strings represent the corresponding keys used to decode hidden information in a response system based on Au−Ag−Cr NCs.

**
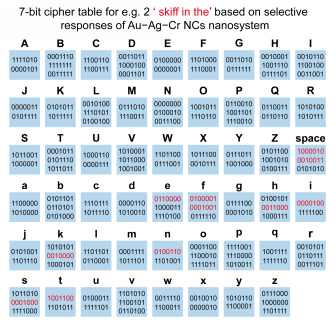
**

**Figure S37.** 7-bit cipher table for e.g. 2 ‘ skiff in the’ based on selective responses (ΔA_416 nm_ *ΔA_530 nm_)

of Au−Ag−Cr NCs nanosystem to metal ions for encoding 53 printable ASCII characters. The colored 7-bit binary strings represent the corresponding keys used to decode hidden information in a response system based on Au−Ag−Cr NCs.

**
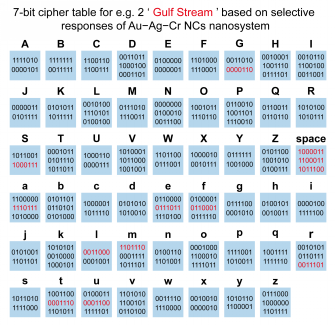
**

**Figure S38.** 7-bit cipher table for e.g. 2 ‘ Gulf Stream ’ based on selective responses (ΔA_416 nm_ *ΔA_700 nm_) of Au−Ag−Cr NCs nanosystem to metal ions for encoding 53 printable ASCII characters. The colored 7-bit binary strings represent the corresponding keys used to decode hidden information in a response system based on Au−Ag−Cr NCs.

**
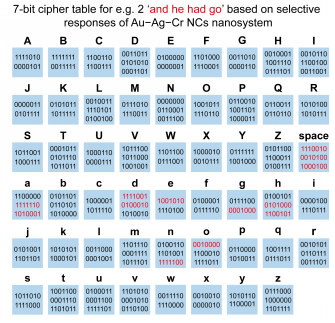
**

**Figure S39.** 7-bit cipher table for e.g. 2 ‘and he had go’ based on selective responses (ΔA_530 nm_ *ΔA_700 nm_) of Au−Ag−Cr NCs nanosystem to metal ions for encoding 53 printable ASCII characters. The colored 7-bit binary strings represent the corresponding keys used to decode hidden information in a response system based on Au−Ag−Cr NCs.

**
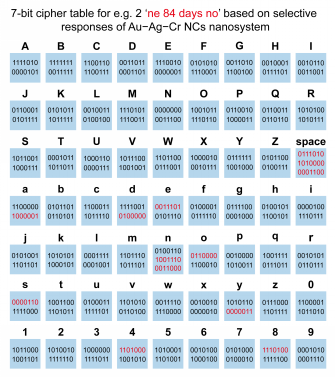
**

**Figure S40.** 7-bit cipher table for e.g. 2 ‘ne 84 days no’ based on selective responses (SUM ΔA_(328-500 nm)_) of Au−Ag−Cr NCs nanosystem to metal ions for encoding 63 printable ASCII characters. The colored 7-bit binary strings represent the corresponding keys used to decode hidden information in a response system based on Au−Ag−Cr NCs.

**
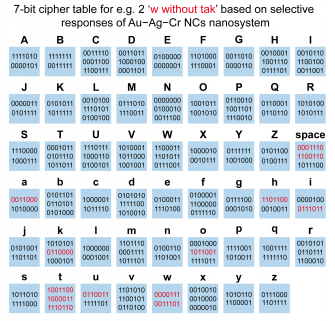
**

**Figure S41.** 7-bit cipher table for e.g. 2 ‘w without tak’ based on selective responses (SUM ΔA_(502-700 nm)_) of Au−Ag−Cr NCs nanosystem to metal ions for encoding 53 printable ASCII characters. The colored 7-bit binary strings represent the corresponding keys used to decode hidden information in a response system based on Au−Ag−Cr NCs.

**
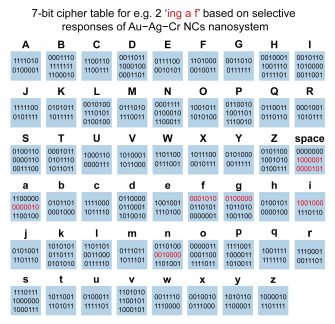
**

**Figure S42.** 7-bit cipher table for e.g. 2 ‘ing a f’ based on selective responses (ΔA_416 nm_) of Au−Ag−Cr NCs nanosystem to anions or reducing agents for encoding 53 printable ASCII characters. The colored 7-bit binary strings represent the corresponding keys used to decode hidden information in a response system based on Au−Ag−Cr NCs.

**
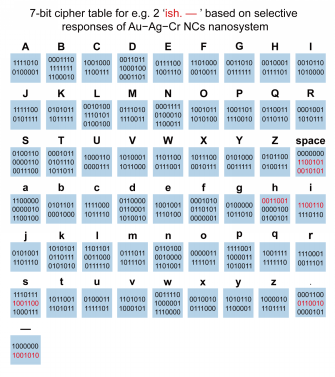
**

**Figure S43.** 7-bit cipher table for e.g. 2 ‘ish. − ’ based on selective responses (ΔA_530 nm_) of Au−Ag−Cr NCs nanosystem to anions or reducing agents for encoding 55 printable ASCII characters. The colored 7-bit binary strings represent the corresponding keys used to decode hidden information in a response system based on Au−Ag−Cr NCs.

**
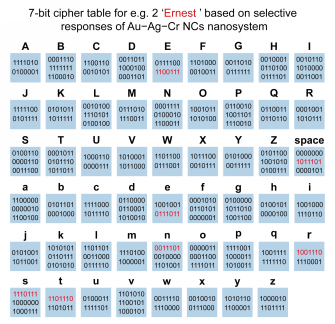
**

**Figure S44.** 7-bit cipher table for e.g. 2 ‘Ernest ’ based on selective responses (ΔA_700 nm_) of Au−Ag−Cr NCs nanosystem to anions or reducing agents for encoding 53 printable ASCII characters. The colored 7-bit binary strings represent the corresponding keys used to decode hidden information in a response system based on Au−Ag−Cr NCs.

**
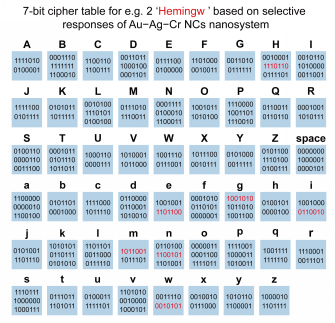
**

**Figure S45.** 7-bit cipher table for e.g. 2 ‘Hemingw ’ based on selective responses (ΔA_416 nm_ *ΔA_530 nm_) of Au−Ag−Cr NCs nanosystem to anions or reducing agents for encoding 53 printable ASCII characters. The colored 7-bit binary strings represent the corresponding keys used to decode hidden information in a response system based on Au−Ag−Cr NCs.

**
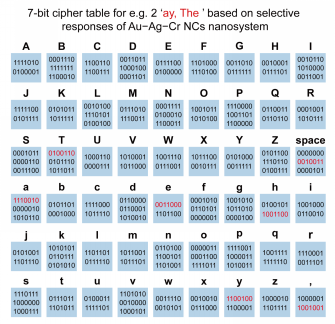
**

**Figure S46.** 7-bit cipher table for e.g. 2 ‘ay, The ’ based on selective responses (ΔA_416 nm_ *ΔA_700 nm_) of Au−Ag−Cr NCs nanosystem to anions or reducing agents for encoding 54 printable ASCII characters. The colored 7-bit binary strings represent the corresponding keys used to decode hidden information in a response system based on Au−Ag−Cr NCs.

**
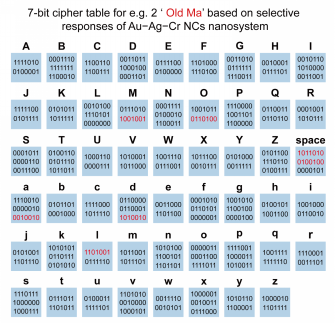
**

**Figure S47.** 7-bit cipher table for e.g. 2 ‘Old Ma’ based on selective responses (ΔA_530 nm_ *ΔA_700 nm_) of Au−Ag−Cr NCs nanosystem to anions or reducing agents for encoding 53 printable ASCII characters. The colored 7-bit binary strings represent the corresponding keys used to decode hidden information in a response system based on Au−Ag−Cr NCs.

**
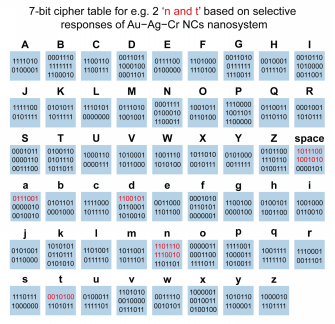
**

**Figure S48.** 7-bit cipher table for e.g. 2 ‘n and t’ based on selective responses (SUM ΔA_(328-500 nm)_) of Au−Ag−Cr NCs nanosystem to anions or reducing agents for encoding 53 printable ASCII characters. The colored 7-bit binary strings represent the corresponding keys used to decode hidden information in a response system based on Au−Ag−Cr NCs.

**
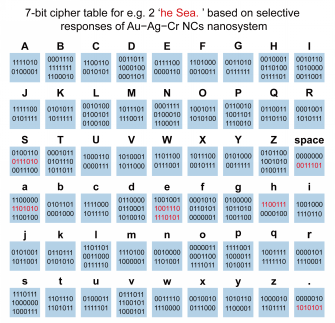
**

**Figure S49.** 7-bit cipher table for e.g. 2 ‘he Sea.’ based on selective responses (SUM ΔA_(502-700 nm)_) of Au−Ag−Cr NCs nanosystem to anions or reducing agents for encoding 54 printable ASCII characters. The colored 7-bit binary strings represent the corresponding keys used to decode hidden information in a response system based on Au−Ag−Cr NCs.

**
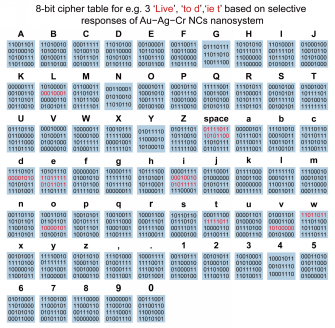
**

**Figure S50.** The first set of 8-bit cipher table for line 1-4 based on molecular logic operations of Au−Ag−Cr nanosystem, used to encode 65 printable ASCII characters. The red fonts represent the corresponding encryption key for decoding the message ‘Live’, ‘to d’, ‘ie t’ of the Au−Ag−Cr NCs logic system.

**
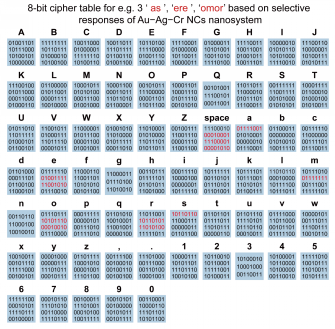
**

**Figure S51.** The second set of 8-bit cipher table for line 5-8 based on molecular logic operations of Au−Ag−Cr nanosystem, used to encode 65 printable ASCII characters. The red fonts represent the corresponding encryption key for decoding the message ‘ as ’, ‘ere ’, ‘omor’ of the Au−Ag−Cr NCs logic system.

**
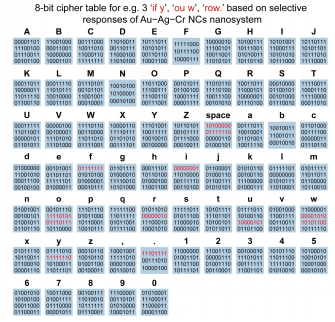
**

**Figure S52.** The third set of 8-bit cipher table for line 9-12 based on molecular logic operations of Au−Ag−Cr nanosystem, used to encode 65 printable ASCII characters. The red fonts represent the corresponding encryption key for decoding the message ‘if y’, ‘ou w’, ‘row.’ of the Au−Ag−Cr NCs logic system.

**References**

(1) Xing, T.-Y.; Zhao, J.; Weng, G.-J.; Li, J.-J.; Zhu, J.; Zhao, J.-W. Synthesis of dual-functional Ag/Au nanoparticles based on the decreased cavitating rate under alkaline conditions and the colorimetric detection of mercury(ii) and lead(ii). *Journal of Materials Chemistry C* **2018**, *6* (28), 7557-7567.

(2) Zhang, J.; Zhu, K.; Hao, H.; Huang, G.; Gan, W.; Wu, K.; Zhang, Z.; Fu, X. A novel chitosan modified Au@Ag core-shell nanoparticles sensor for naked-eye detection of Hg2+. *Materials Research Express* **2019**, *6* (12), 125045.

(3) Zhu, J.; Zhao, B.-z.; Qi, Y.; Li, J.-J.; Li, X.; Zhao, J.-W. Colorimetric determination of Hg(II) by combining the etching and aggregation effect of cysteine-modified Au-Ag core-shell nanorods. *Sensors and Actuators B: Chemical* **2018**, *255*, 2927-2935.

(4) Chen, N.; Zhang, Y.; Liu, H.; Wu, X.; Li, Y.; Miao, L.; Shen, Z.; Wu, A. High-Performance Colorimetric Detection of Hg2+ Based on Triangular Silver Nanoprisms. *ACS Sensors* **2016**, *1* (5), 521-527.

(5) Ma, C.; Ma, Y.; Sun, Y.; Lu, Y.; Tian, E.; Lan, J.; Li, J.; Ye, W.; Zhang, H. Colorimetric determination of Hg2+ in environmental water based on the Hg2+-stimulated peroxidase mimetic activity of MoS2-Au composites. *Journal of Colloid and Interface Science* **2019**, *537*, 554-561.

(6) Amirjani, A.; Haghshenas, D. F. Facile and on−line colorimetric detection of Hg2+based on localized surface plasmon resonance (LSPR) of Ag nanotriangles. *Talanta* **2019**, *192*, 418-423.

(7) Hu, J.; Liu, T.; Gao, H.-W.; Lu, S.; Uvdal, K.; Hu, Z. Selective detections of Hg2+ and F− by using tailor-made fluorogenic probes. *Sensors and Actuators B: Chemical* **2018**, *269*, 368-376.

(8) Bozkurt, E.; Gul, H. I. A novel pyrazoline-based fluorometric “turn-off” sensing for Hg2+. *Sensors and Actuators B: Chemical* **2018**, *255*, 814-825.

(9) Xu, S.; Liu, Y.; Yang, H.; Zhao, K.; Li, J.; Deng, A. Fluorescent nitrogen and sulfur co-doped carbon dots from casein and their applications for sensitive detection of Hg2+ and biothiols and cellular imaging. *Analytica Chimica Acta* **2017**, *964*, 150-160.

(10) Yang, H.; Peng, C.; Han, J.; Song, Y.; Wang, L. Three-dimensional macroporous Carbon/Zr-2,5-dimercaptoterephthalic acid metal-organic frameworks nanocomposites for removal and detection of Hg(II). *Sensors and Actuators B: Chemical* **2020**, *320*, 128447.

(11) Wei, Z. N.; Li, H. Q.; Liu, S. B.; Wang, W.; Chen, H. L.; Xiao, L. H.; Ren, C. L.; Chen, X. G. Carbon Dots as Fluorescent/Colorimetric Probes for Real-Time Detection of Hypochlorite and Ascorbic Acid in Cells and Body Fluid. *Analytical Chemistry* **2019**, *91* (24), 15477-15483.

(12) Walekar, L. S.; Pawar, S. P.; Gore, A. H.; Suryawanshi, V. D.; Undare, S. S.; Anbhule, P. V.; Patil, S. R.; Kolekar, G. B. Surfactant stabilized AgNPs as a colorimetric probe for simple and selective detection of hypochlorite anion (ClO<SUP>-</SUP>) in aqueous solution: Environmental sample analysis. *Colloids and Surfaces a-Physicochemical and Engineering Aspects* **2016**, *491*, 78-85.

(13) Zhu, B. C.; Xu, Y. H.; Liu, W. Q.; Shao, C. X.; Wu, H. F.; Jiang, H. L.; Du, B.; Zhang, X. L. A highly selective colorimetric probe for fast and sensitive detection of hypochlorite in absolute aqueous solution. *Sensors and Actuators B-Chemical* **2014**, *191*, 473-478.

(14) Lv, B. Y.; Wang, Z. L.; Wu, Y. S.; Zheng, Y. M.; Cui, Z. N.; Li, J.; Gu, W. A novel dual-responsive colorimetric/fluorescent probe for the detection of N<sub>2</sub>H<sub>4</sub> and ClO<SUP>-</SUP> and its application in environmental analysis and bioimaging. *Journal of Hazardous Materials* **2024**, *469*.

(15) Li, X. X.; Lin, X.; Lin, S.; Sun, X. C.; Gao, D.; Liu, B. K.; Zhao, H. Y.; Zhang, J.; Cong, S. L.; Wang, L. Au Nanospheres@Ag Nanorods for Wide Linear Range Colorimetric Determination of Hypochlorite. *Acs Applied Nano Materials* **2019**, *2* (5), 3161-3168.

(16) Gil, D.; Choi, B.; Lee, J. J.; Lee, H.; Kim, K. T.; Kim, C. A colorimetric/ratiometric chemosensor based on an aggregation-induced emission strategy for tracing hypochlorite in vitro and in vivo. *Ecotoxicology and Environmental Safety* **2023**, *257*.

(17) Yan, F.; Bai, Z.; Ma, T.; Sun, X.; Zu, F.; Luo, Y.; Chen, L. Surface modification of carbon quantum dots by fluorescein derivative for dual-emission ratiometric fluorescent hypochlorite biosensing and in vivo bioimaging. *Sensors and Actuators B: Chemical* **2019**, *296*, 126638.

(18) Du, Y.; Wang, B.; Jin, D.; Li, M.; Li, Y.; Yan, X.; Zhou, X.; Chen, L. Dual-site fluorescent probe for multi-response detection of ClO(-) and H(2)O(2) and bio-imaging. *Anal Chim Acta* **2020**, *1103*, 174-182.

(19) Tan, H. L.; Wu, X. Y.; Weng, Y. H.; Lu, Y. J.; Huang, Z. Z. Self-Assembled FRET Nanoprobe with Metal-Organic Framework As a Scaffold for Ratiometric Detection of Hypochlorous Acid. *Analytical Chemistry* **2020**, *92* (4), 3447-3454.

(20) Gu, Y. T.; Zheng, X. Y.; Chen, Z. K.; Teng, R. M.; Zhang, Y. H.; Li, H.; Ding, C. P.; Huang, Y. J. Fluorescent-colorimetric dual signal ratio sensor with AuNRs@UCNPs superstructure nanoprobe for accurate hypochlorite detection. *Sensors and Actuators B-Chemical* **2024**, *419*.
